# Vascular Endothelial Growth Factor (VEGF) Induced Downstream Responses to Transient Receptor Potential Vanilloid 1 (TRPV1) and 3-Iodothyronamine (3-T_1_AM) in Human Corneal Keratocytes

**DOI:** 10.3389/fendo.2018.00670

**Published:** 2018-11-22

**Authors:** Ersal Türker, Fabian Garreis, Noushafarin Khajavi, Peter S. Reinach, Pooja Joshi, Tobias Brockmann, Alexander Lucius, Nina Ljubojevic, Elizabeth Turan, Drew Cooper, Felix Schick, Rob Reinholz, Uwe Pleyer, Josef Köhrle, Stefan Mergler

**Affiliations:** ^1^Klinik für Augenheilkunde, Charité—Universitätsmedizin Berlin, Corporate Member of Freie Universität Berlin, Humboldt-Universität zu Berlin, and Berlin Institute of Health, Berlin, Germany; ^2^Department of Functional and Clinical Anatomy, Friedrich Alexander University Erlangen-Nürnberg, Erlangen, Germany; ^3^Institut für Experimentelle Pädiatrische Endokrinologie, Charité—Universitätsmedizin Berlin, Corporate Member of Freie Universität Berlin, Humboldt-Universität zu Berlin, and Berlin Institute of Health, Berlin, Germany; ^4^Walter Straub Institute of Pharmacology and Toxicology, Ludwig Maximilian University of Munich, Munich, Germany; ^5^School of Ophthalmology and Optometry, Wenzhou Medical University, Wenzhou, China; ^6^Berlin Institute of Health (BIH), Berlin, Germany; ^7^Institut für Experimentelle Endokrinologie, Charité—Universitätsmedizin Berlin, Corporate Member of Freie Universität Berlin, Humboldt-Universität zu Berlin, and Berlin Institute of Health, Berlin, Germany

**Keywords:** human corneal keratocytes, 3-iodothyronamine, vascular endothelial growth factor, transient receptor potential channel vanilloid 1, transient receptor potential channel melastatin 8, intracellular Ca^2+^, planar patch-clamp technique, thyronamine

## Abstract

This study was undertaken to determine if crosstalk among the transient receptor potential (TRP) melastatin 8 (TRPM8), TRP vanilloid 1 (TRPV1), and vascular endothelial growth factor (VEGF) receptor triad modulates VEGF-induced Ca^2+^ signaling in human corneal keratocytes. Using RT-PCR, qPCR and immunohistochemistry, we determined TRPV1 and TRPM8 gene and protein coexpression in a human corneal keratocyte cell line (HCK) and human corneal cross sections. Fluorescence Ca^2+^ imaging using both a photomultiplier and a single cell digital imaging system as well as planar patch-clamping measured relative intracellular Ca^2+^ levels and underlying whole-cell currents. The TRPV1 agonist capsaicin increased both intracellular Ca^2+^ levels and whole-cell currents, while the antagonist capsazepine (CPZ) inhibited them. VEGF-induced Ca^2+^ transients and rises in whole-cell currents were suppressed by CPZ, whereas a selective TRPM8 antagonist, AMTB, increased VEGF signaling. In contrast, an endogenous thyroid hormone-derived metabolite 3-Iodothyronamine (3-T_1_AM) suppressed increases in the VEGF-induced current. The TRPM8 agonist menthol increased the currents, while AMTB suppressed this response. The VEGF-induced increases in Ca^2+^ influx and their underlying ionic currents stem from crosstalk between VEGFR and TRPV1, which can be impeded by 3-T_1_AM-induced TRPM8 activation. Such suppression in turn blocks VEGF-induced TRPV1 activation. Therefore, crosstalk between TRPM8 and TRPV1 inhibits VEGFR-induced activation of TRPV1.

## Introduction

Numerous different transient receptor potential (TRP) nonselective ionic channel subtypes are functionally expressed on nearly every tissue in the human body. They act as polymodal sensors of environmental stresses and induce both adaptive and maladaptive responses under different conditions (1–3). In this regard, TRP channels play an essential role in inflammation, neovascularization, fibrosis, and pain perception (4–7). The 28 mammalian TRP channel subtypes are subdivided into six main subfamilies based on differences in their amino acid sequence homology (8). They display greater diversity in activation mechanisms and selectivity than any other group of ion channels. TRP vanilloid type 1 (TRPV1; capsaicin receptor) belongs to the vanilloid subfamily and is an archetype for other TRP ion channels that transduce a host of environmental stresses to elicit both adaptive and maladaptive responses (9).

TRPV1 is expressed in different ocular tissues including the corneal epithelium and endothelium (10–12), as well as stromal fibroblasts (13) and conjunctival epithelial cells (HCjEC) (14). Its activation in the cornea has cell type specific effects that can either promote restoration or impair recovery of corneal function subsequent to injury. Due to its central role in corneal wound healing, such functional diversity indicates the need to expand the current understanding of mechanisms involved in modulating TRPV1 activation (14–16). Subsequent to corneal epithelial delimited injury, TRPV1 activation contributes to mediating the increases in cell proliferation and migration induced by epidermal growth factor (EGF) (17, 18). On the other hand, exposure to hypertonic stress induces increases in pro-inflammatory cytokine expression through TRPV1 activation in human corneal epithelial cells (HCEC) (19). If such an effect is self-limiting, it can be of adaptive value in protecting the cornea from pathogenic infiltration. However, if the response does not resolve itself such an effect can be maladaptive resulting in inflammatory fibrosis and opacification (37). Similarly, stromal cell TRPV1 upregulation and activation by a severe stromal injury leads to inflammatory fibrosis as a result of crosstalk with transforming growth factor β-1 (TGF β-1) (38). In addition to inflammatory fibrosis, stromal injury can lead to neovascularization arising from increases in vascular endothelial growth factor (VEGF) gene and protein expression (39). Even though activation of Ca^2+^ signaling contributes to this response to VEGF, it is unclear if neovascularization is affected by crosstalk between VEGFR and TRPV1.

Functional TRP melastatin type 8 (TRPM8) expression has been characterized on corneal nerves and endothelial cells (11, 40, 41) as well as corneal epithelial cells (42) and conjunctival epithelial cells (14, 43). Both TRPV1 and TRPM8 are thermosensitive channels whose activity is modulated by temperature changes encountered in daily living. TRPM8 can be activated by moderate cooling from ~25–28°C, as well as by menthol and icilin (44, 45), whereas TRPV1 increases its activity starting at 43°C (46).

3-Iodothyronamine (3-T_1_AM) is a thyroid hormone metabolite and was detected in rodent tissues (47) and in human serum (48). This endogenous amine stimulates TRPM8 activity, which in turn suppresses TRPV1 activity in thyroid cells as well as conjunctival epithelial and corneal epithelial and endothelial cells (49, 50). Administering high doses of 3-T_1_AM *in vivo* induces profound hypothermia within minutes in mice and hamsters (20), whereas lower doses do not evoke hypothermia but instead other reactions (51). Recent studies clarified some mechanisms and propose anapyrexia and vasodilation instead of hypothermia (52). Interestingly, TRPM8 activation by this agonist inhibited rises in TRPV1 activity induced by capsaicin in HCEC and HCjEC (42, 43).

Even though the cognate VEGF receptor (VEGFR) and TRPV1 are coexpressed on corneal fibroblasts (53), it is unclear if the Ca^2+^ transients mediating VEGF-induced stromal angiogenesis stem from VEGFR-mediated activation of TRPV1 (13, 54, 55). A clear indication that neovascularization and the increased expression of VEGF following corneal chemical injury may depend on TRPV1 expression, is that those tissue responses were markedly attenuated in TRPV1 knockout mice (53). Besides neovascularization, VEGF upregulation contributes to increased endothelial cell proliferation and migration in a bovine wound healing model (56, 57). Furthermore, it is also unknown if TRPM8 activation alters VEGF-induced TRPV1 activation.

In this study, we document that TRPM8 activation suppresses TRPV1 responsiveness toward VEGFR activation. Such disruption of VEGFR-induced TRPV1 activation suggests that TRPM8 is potentially a viable target for the suppression of TRPV1-induced inflammatory fibrosis and neovascularization, which may also be of clinical relevance.

## Materials and methods

### Medium and reagents

The *K*eratinocyte *B*asal *M*edium KBM® provided by Lonza (Köln, Germany) is referred to as the *K*eratinocyte *G*rowth *M*edium KGM®. VEGF-165 human recombinant protein was purchased from ThermoFisher Scientific (Rockford, IL, USA). Icilin was obtained from the Cayman Chemical Company (Ann Arbor, Michigan, USA). BCTC and AMTB were purchased from TOCRIS Bioscience (Bristol, UK). Accutase was purchased from PAA Laboratories (Pasching, Austria). All other reagents were purchased from Sigma-Aldrich (Darmstadt, Germany).

### Corneas

Human corneas were obtained from cadavers (two males, three females, average age 75 ± 5 years). Donors provided written consent to the Department of Anatomy, Friedrich Alexander University Erlangen-Nürnberg (FAU), Germany. They were free of recent ocular surface trauma, eye infections or diseases. All corneas were dissected within 4 h and up to 24 h post-mortem.

Primary human cornea fibroblast cells (pHCF) were obtained from corneal peripheral remnants of transplants donated to German eye banks. Donors signed a patient consent form and the study complied with the underlying legal requirements and with the Helsinki Declaration. Epithelium was removed mechanically under a stereo microscope. Corneal stroma was cut into pieces (diameter of ≈1 mm), placed on 6-well petri dish and cultivated with Dulbecco's modified Eagle's medium (DMEM) HAMS F-12 (Biochrome AG, Berlin, Germany) containing 10% fetal bovine serum (FBS, Thermo Fisher Scientific, Waltham, USA). The medium was replaced at least every second day and primary cell culture (passage 0) was split with trypsin at a density of 80%. Cells were cultivated at 37°C in a humidified 5% CO_2_ incubator. For the present analysis, 8 different remnants of corneal transplantation from passage 1 to 5 were used.

### Cell culture of HCK

SV40-immortalized human corneal keratocytes (HCK) were kindly provided by Michaela Zorn-Kruppa et al. from the Eppendorf-Hospital in Hamburg, Germany and cultivated according to their protocols (30, 58, 59). For the HCK cultivation, the same protocol was used as for the aforementioned pHCF cells. In brief, cells were grown in KGM® containing recombinant human epidermal growth factor (rhEGF 0.1 ng/ml), hydrocortisone (≈0.5 μg/ml), insulin (≈5 μg/ml), bovine pituitary extract (BPE) and 0.5 mM calcium chloride (CaCl_2_) as well as penicillin/streptomycin in a humidified 5% CO_2_ incubator at 37°C (59). For the electrophysiological measurements, cell confluence ranged between 50 and 80%. At these different degrees of confluence, their electrophysiological characteristics were invariant (60).

### RNA isolation and RT-PCR

HCK, HCEC-12 and LNCaP (human prostate adenocarcinoma) cells were seeded in T75 flasks and grown to 80% confluence. Cells were harvested following three different passages. Total RNA was extracted using TRIzol® Reagent RT (Ambion, Austin, TX) according to manufacturer's instructions. The quality of extracted RNA was evaluated by NanoDrop ND-2000 spectrophotometer (PEQLAB, Germany. DNase digestion was performed and samples were stored at −80°C. RNA (2 μg) was transcribed into cDNA by high capacity cDNA reverse transcription kit (Applied Biosystems, Darmstadt, Germany). cDNA was denatured (95°C, 5 min), first strand synthesized (42°C, 50 min) and the reaction was terminated by heating to 70°C for 15 min. For RT-PCR, 2 μl cDNA mixture was used as a template in subsequent amplification reactions in a total of 30 μl volume containing specific primers for TRPM8 (sequences Fwd: CCTGTTCCTCTTTGCGGTGTGGAT; Rev: TCCTCTGAGGTGTCGTTGGCTTT) generating a 621-bp product. Glyceraldehyde-3-phosphate dehydrogenase (GAPDH) was used as control (sequences Fwd: TCAACGACCACTTTGTCAAGCTCA; Rev: GCTGGTGGTCCAGGGGTCTTACT) generating an anticipated 119-bp product. Each reaction also contained red PCR Master Mix (Stratec Biomedical AG, Birkenfeld, Germany). PCR reaction underwent the first cycle at 95°C for 5 min, followed by 35 cycles of a repeat of denaturation at 95°C for 15 s, a primer-specific annealing step at 58°C for 30 s (TRPM8) or 60°C for 30 s (GAPDH) and a primer-specific elongation step at 72°C for 45 s (GAPDH) or at 72°C for 7 min (TRPM8), followed by a final temperature holding at 4°C. Eight microliters of the PCR products were loaded on a 1.5% agarose gel and after electrophoresis they were visualized via ethidium bromide staining under UV light.

### Quantitative RT-PCR

TRPM8 specific primers (sequences Fwd: ATGGCCGGGACGAGATGGACA; Rev: AGCCCCTGGTCTGCTCCCAAA) generated 138-bp products. Aforementioned GAPDH was also used as the reference gene in qPCR. Amplification was carried out using the Mx3000P qPCR system real-time cycler (Stratagene, Waldbronn, Germany). For detection LightCycler® 480 SYBR Green I Master (Roche, Germany) was used. Amplification was performed for 45 cycles lasting 15 s (95°C) and 30 s (60°C) as previously described (15, 43). GAPDH expression levels normalized TRPM8 gene expression levels. Melting curve analysis was performed to confirm the specificity of the PCR reaction. Data was processed using double delta Ct analysis. Each of the three passages of every cell line was measured in triplicate together with a sample without reverse transcription to exclude genomic DNA contamination.

### Immunocytochemistry

Human corneas were fixed in 4% paraformaldehyde (Roth, Karlsruhe, Germany) for 4 h. After washing with PBS, they were mounted in tissue-freezing medium (Leica, Wetzlar, Germany) and 10 μm thick sections were prepared. They were incubated in Blotto blocking buffer (Thermo Scientific, Waltham, Massachusetts, USA) at RT for 1 h and afterwards with anti-TRPM8 (1:50, HPA024117, Sigma-Aldrich, St. Louis, Missouri, USA) or anti-TRPV1 (1:50, ACC-030, Alomone, Jerusalem, Israel) antibody at 4°C overnight. After washing three times with PBS, sections were incubated with a secondary Alexa Fluor 488-labeled antibody (1:1000, A11070, Life Technologies, Carlsbad, CA, USA) at RT for 1 h. The cell nuclei were counter-stained with 4'6-diamidino-2-phenylindole (DAPI D9564, Sigma-Aldrich, St. Louis, MO, USA) for 10 min. After washing with PBS, sections were embedded with fluorescence mounting medium (S3023, Dako, Glostrup, Denmark) and stored at 4°C in the dark. Antibody specificity was confirmed as previously described (16). All slides were examined with a Keyence Biorevo BZ9000 microscope (Keyence, Neu-Isenburg, Germany). HCK and pHCF seeded on glass coverslips were maintained at 37°C in a humidified 5% CO_2_ incubator until they were 50–70% confluent. Then they were fixed on ice in 4% (w/v) paraformaldehyde for 20 min and rinsed twice with PBS and permeabilized with Triton X-100 (0.1%). For HCK TRPV1/TRPM8 and biomarker detection, methanol fixed cells for 10 min at −20°C were used. Non-specific antibody binding was blocked with 1% BSA. Cells were then incubated overnight at 4°C with a rabbit anti-TRPM8 monoclonal antibody (1:1000, Abcam, Cambridge, UK), anti-α-SMA (1:500, EPR5368, Epitomics, Burlingame, CA, USA) (35, 36), anti-lumican (1:100, AF2846; R&D System, Minneapolis, MN, USA) or anti-keratocan antibody (1:100, ab128304; Abcam, Cambridge, UK) (32, 61). After washing twice with PBS, they were then exposed to a secondary fluorescence-labeled antibody for 1 h and mounted with DAPI for 5 min. For fluorescence visualization, a Zeiss AxioImager M2 inverted microscope (Zeiss, Oberkochen, Germany) was used. HCK cells were cultured 3 days in 12-well cell culture plates. Vital cells were fixed for 30 min using 4% formaldehyde, followed by washing three times with Tris-buffered saline (TBS; pH 7.6, 10 min each). Cells were permeabilized with 0.1% Triton (15 min) and blocked with 5% BSA in TBS for 60 min. HCK cells were incubated overnight in a humidified chamber at 4°C with primary CD90 antibodies (1:200, ab181469, Abcam, Cambridge, UK) (25) diluted in 0.8% BSA in TBS. Fluorescence detection employed fluorescein isothiocyanate conjugated secondary antibodies (1:200, F8771; Sigma-Aldrich, St. Louis, Missouri, USA) and DAPI nuclear counterstaining. Mounted slides were examined using light microscopy (Axio Imager.M2; Zeiss, Jena, Germany).

### CD90 paraffin embedded immunohistochemistry in human corneal stroma (pIHC)

A corneal cross section obtained from a patient with corneal stromal fibrosis during perforating keratoplasty was used as a positive control. Sections of this 4% formaldehyde fixed paraffin embedded tissue were incubated at 60°C (60 min), deparaffinized with xylol (40 min) followed by isopropanol (30 min) and rehydrated through a descending alcohol series (96, 90, 70, and 50% alcohol, 5 min each) to 0.3% hydrogen peroxide in aqua (5 min) and TPB (pH 7.6, 5 min). Antigen retrieval was performed with 0.1% trypsin for 60 min. Tissue sections were permeabilized with 0.1% Triton X-100 (10 min) and blocked with 5% BSA in TBS for 60 min. These tissue sections were incubated overnight in a humidified chamber at 4°C with primary antibodies diluted in 0.8% BSA in TBS against CD90 (1:30, ab181469, Abcam, Cambridge, UK). Fluorescence detection employed fluorescein isothiocyanate conjugated secondary antibodies (1:100, F8771; Sigma-Aldrich, St. Louis, Missouri, USA) and DAPI nuclear counterstaining. Mounted slides were examined using light microscopy (Axio Imager.M2; Zeiss, Jena, Germany).

### Fluorescence calcium imaging

HCK cells were cultivated on 15 mm diameter glass cover slips placed in a culture plate with wells until they reached a semi confluent stage (≈60–80%). The culture conditions used to prepare cells for immunofluorescence were the same as those used for Ca^2+^ imaging. The cells were loaded with fura-2/AM (1 μM) at 37°C for 20–40 min. Loading was stopped with a Ringer-like (control) solution containing (mM): 150 NaCl, 6 CsCl, 1 MgCl_2_, 10 glucose, 10 HEPES, and 1.5 CaCl_2_ at pH 7.4 and 317 mOsM (62). Potassium was replaced by cesium and the coverslips were placed on an inverted microscope stage (Olympus BW50WI, Olympus Europa Holding GmbH, Hamburg, Germany) inside an experimental chamber containing the same solution. This setup was connected to a digital imaging system (TILL Photonics, Munich, Germany), outfitted for UV excitation. Fura-2/AM fluorescence was alternately excited at 340 nm and 380 nm and monitored for different times at 500 ms intervals (63). The corresponding emission wavelength was 510 nm. The fluorescence ratio (f_340nm_/f_380nm_) was calculated by the software. This ratio is a relative index of intracellular Ca^2+^ ([Ca^2+^]_i_) levels (63). The f_340nm_/f_380nm_ dynamic ratio range was small [0.2 (42)] because single fluorescence response signals at 340 and 380 nm were set to a fixed value to avoid distortions of the Ca^2+^ responses patterns. A TRPM8 control was provided by overexpressing TRPM8 in transfected cells and exposing them to described agonists (42). The measuring field was adapted to the number of cells (TILL Photonics view finding system). Before each experiment, cells were routinely tested to determine whether the calcium homeostasis (control baseline) remained constant for 10 min. The control measurements are shown with open circles in the figures. All experiments were performed at a constant room temperature (≈23°C). If stabilization had not occurred within the first 5 min, adaptation to room temperature was prolonged. Results are shown as mean traces of the f_340nm_/f_380nm_ ratio ± SEM (error bars in both directions) with n*-*values indicating the number of experiments per data point. For 10 min, measurements were obtained from groups of 5–10 cells at least three times. The fluorescence ratios were normalized (control set to 1.2) and averaged (with error bars). In addition, the fura-2-induced fluorescence signals were alternatively acquired and evaluated using Life Science fluorescence cell imaging software “cellSens” (Olympus, Hamburg, Germany) in conjunction with a digital camera (Olympus XM-10) (Figures 4G,I, 5D,F, 8F,H, 10E,I; control set to 0.1 and 0.2, resp.). For the fluorescence excitation wavelengths (340 and 380 nm), specific filters and a LED light source were used (LED-Hub by Omikron, Rodgau-Dudenhoven, Germany). Fura-2 fluorescence was alternately excited at 340 and 380 nm and emission was detected every 5 s at 510 nm (250–3,800 ms exposure time). The relatively slow rise times of Ca^2+^ transients in all experiments were a consequence of drugs being pipetted into a stationary bath rather than a flow through system. In such a configuration, the time delay was 1–2 min between drug addition and peak response. When using a blocker, cells were preincubated with the blocker for ~30 min before the measurement and all test solutions contained the blocker. Drugs were dissolved in dimethyl sulfoxide (DMSO) to obtain a stock solution and diluted to provide a working concentration that did not exceed 0.1%. This DMSO concentration was nontoxic based on stable f_340nm_/f_380nm_ levels (data not shown).

### Planar patch-clamp recordings

Whole-cell currents were measured using a planar patch-clamp setup (Port-a-Patch®, Nanion, Munich, Germany) in conjunction with an EPC 10 patch-clamp amplifier (HEKA, Lamprecht, Germany) and controlled by PatchMaster software (Version 2.6; HEKA, Lamprecht, Germany). A standard intracellular solution containing (mM): 50 CsCl, 10 NaCl, 60 CsF, 20 EGTA, and 10 HEPES at pH ≈7.2 and ≈288 mOsM was applied to the microchip (both provided by Port-a-Patch®, Nanion, Munich, Germany). The external solution contained (mM): 140 NaCl, 4 KCl, 1 MgCl_2_, 2 CaCl_2_, 5 D-glucose monohydrate, and 10 HEPES, pH ≈7.4 and osmolarity ≈298 mOsM. A single cell suspension of 5–10 μl was placed onto a microchip having a 2.5–3 MΩ resistance (aperture ≈1-3 μm). A negative pressure applied by a software-controlled pump (Nanion) fixed one single cell atop the aperture. Mean membrane capacitance (19 ± 2 pF; *n* = 38) and mean access resistance (18 ± 2 MΩ; *n* = 38) were software calculated. Series resistances, fast and slow capacitance transients were compensated by the software of the patch-clamp amplifier. Series resistance did not change markedly during an experiment. The liquid junction potential was calculated (≈3.8 mV) (64), which is in close agreement with the measured range (≈4–7 mV). Its mean value was used to correct the measurements analyzed with the Patch-Master software. Current recordings were all leak-subtracted and cells with leak currents above 100 pA were excluded from analysis. All experiments were performed at 22°C room temperature in an air-conditioned room to avoid confounding responses by other thermo-sensitive TRPs. The current response patterns were generated through application of specific voltage step-protocols, which induced typical TRP channel whole-cell current patterns (62). The holding potential (HP) was set to 0 mV in order to eliminate any possible contribution by voltage-dependent Ca^2+^ channel activity. After confirming the control settings, experiments were started ~10 min after breaking into a whole-cell configuration (65). Whole-cell currents were recorded using 10 mV voltage steps over a range from −60 to +130 mV (10 mV increments) for 400 ms each. Currents were also recorded through a voltage ramp protocol of −60 to +130 mV range and 500 ms duration every 5 s. Resulting currents were normalized with respect to cell membrane capacitance to obtain current density (pA/pF) values displayed in current vs. voltage plots.

### Statistical analysis

Significance was determined using Student's *t*-test for paired data (*p*-values: two-tailed) provided they passed a software available normality test. If the normality test failed, non-parametric Wilcoxon matched pairs were used. For non-paired data, Student's *t*-test for unpaired data was used, if it passed a normality test. Alternatively, the non-parametric Mann-Whitney-U test was performed. Welch's correction was applied if data variance of the two groups were not at the same level. Probabilities of *p* < 0.05 [indicated by asterisks (^*^) and hashtags (#)] were considered to be significant. In addition, normally distributed data of more than two groups were statistically analyzed using one-way ANOVA (one-way analysis of variance). Selected pairs of columns were tested using Bonferroni post-test. If the data were not normally distributed, Kruskal-Wallis test was used and selected pairs of columns were tested using Dunn's post-test. Significance level alpha = 0.05 (95% confidence intervals). The number of repeats is shown in each case in brackets, near the traces or bars. All values are means ± SEM (error bars in both directions). All plots were generated with SigmaPlot software version 12.5 for Windows (Systat Software, San Jose, California, U.S.A.). Bar charts were plotted and statistical analyses were performed using GraphPad Prism (version 5.00 for Windows) (La Jolla, California, USA.).

## Results

### Validation of HCK identity

**Figure 1:**
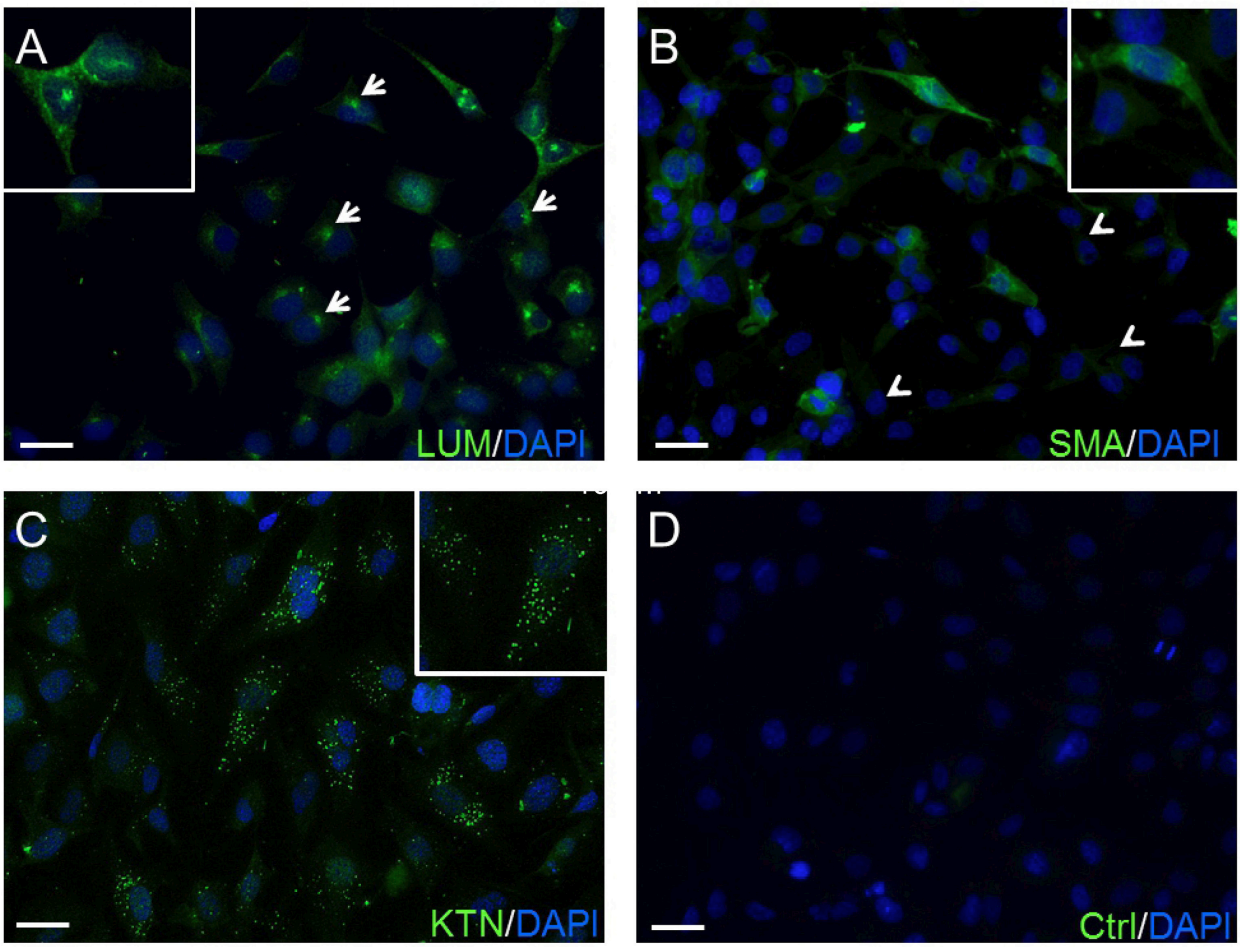
Confirmation of HCK identity. **(A)** The green anti-LUM antibody immunofluorescent (IF) staining is delimited in the endoplasmic reticulum (arrows). **(B)** Anti-α-SMA antibody IF staining pattern. Their green staining pattern is less intense. Approximately 20–30% of the HCK cells have no IF signal (arrow heads). **(C)** IF green staining with anti-KTN antibody detected in all HCKs. **(D)** A negative control (Ctrl) omitted primary antibody and does not provide a specific IF signal. In all experiments, cell nuclei were counterstained with DAPI (blue) and pictures were merged. Pictures are representative of IF results from five different assays (*n* = 5).

**Figure 2:**
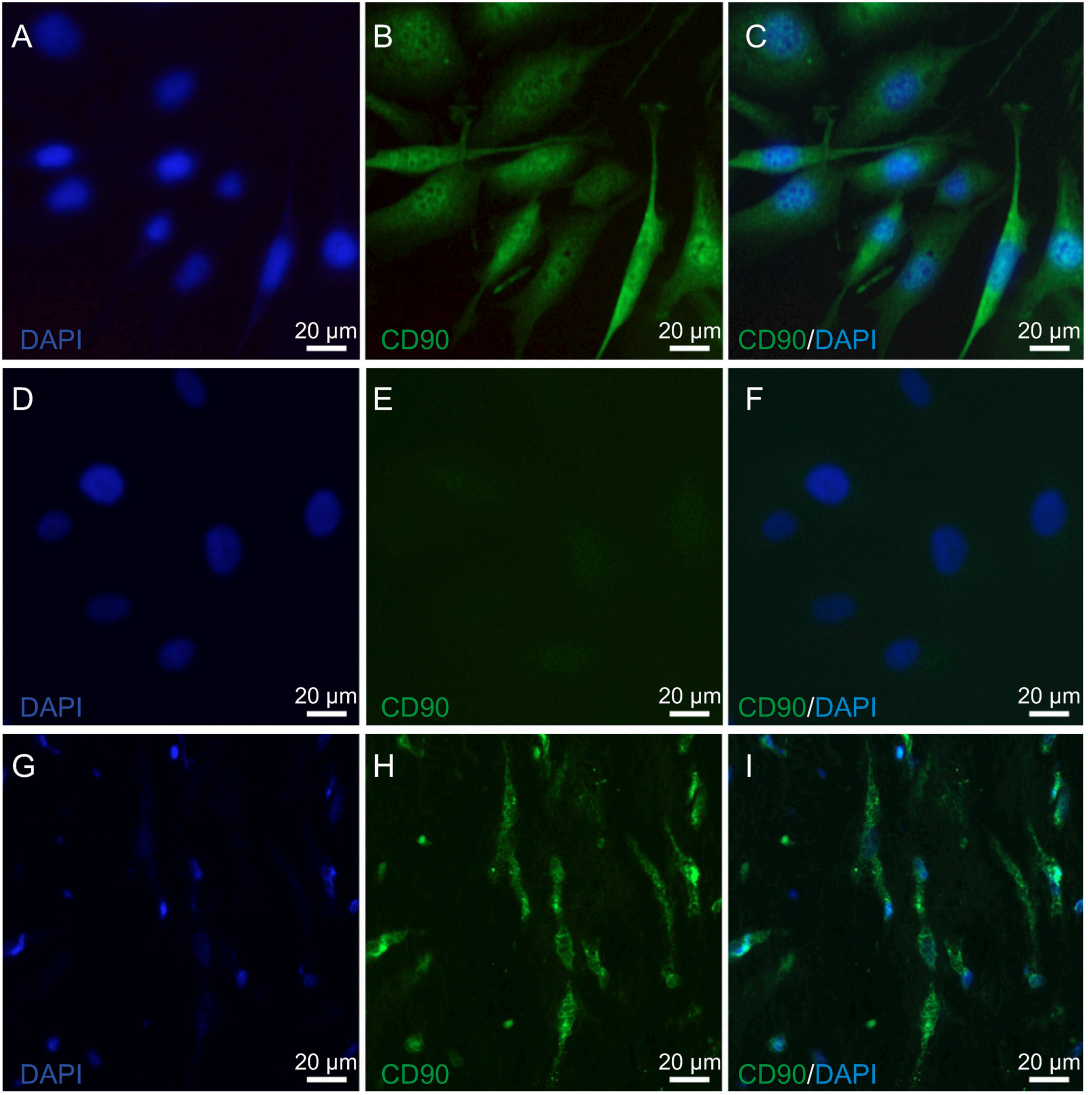
Mesenchymal stem cell identification in HCK and pIHC. **(A)** Nuclear staining of HCK with DAPI (blue). **(B)** Anti CD90 IF antibody green staining detected in HCK. **(C)** Merged B image with DAPI blue staining of cell nuclei. **(D)** DAPI blue staining of nuclei in HCK. **(E)** Omission of anti-CD90 antibody green staining pattern validates primary antibody in HCK. **(F)** Similar as E but with DAPI (blue) (merged). **(G)** Nuclear staining of human corneal stroma (pIHC) with DAPI (blue). **(H)** IF analyses of anti-CD90 antibody green staining in pIHC as a positive control. **(I)** Merged image shown in H panel with DAPI blue counterstaining.

The expression of lumican (LUM), a biomarker of keratocytes, was discernible along with LUM derived green fluorescence, which was localized in the cytoplasm, and also in the endoplasmic reticulum (ER) (66, 67) (Figure 1A). This expression pattern is similar to results obtained with an anti-keratocan (KTN) antibody (Figure 1C), which is a more specific keratocyte marker than LUM. Alpha-smooth muscle actin (α-SMA) expression patterns were different from the patterns with the LUM and KTN biomarker. The α-SMA expression patterns are indicative of myofibroblast coexpression (67). In this case, up to 30% of HCK cells were immunofluorescent (IF). Another 20–30% of these cells displayed a diffuse IF signal whereas ~40–50% of the HCK cells did not express α-SMA (Figure 1B). In controls, omission of the primary antibody eliminated IF (Figure 1D). The specificity of the LUM, KTN and α-SMA antibodies was verified by showing absence of any IF staining in telomerase-immortalized human corneal epithelial (hTCEpi) cells (31). LUM specificity agrees with its failure to stain pHCF cells (data not shown). CD90 is a biomarker of mesenchymal stem cells (MSC) derived from keratocytes *in vitro*. It was also detectable in the cultured stromal cells (HCK) (Figure 2) (68). Immunostaining patterns were eliminated by omitting the primary antibody (Figure 2E). The MSC marker expression panel shows that keratocytes constitute a majority of the cell types in the HCK cell line. They are accompanied by a subpopulation of fibroblasts and myofibroblasts and some mesenchymal stem cells. Such a mixture is unavoidable especially in a serum containing medium since under this condition keratocytes spontaneously undergo transformation acquiring a (myo)fibroblast phenotype (66, 69).

### TRPV1 protein expression

**Figure 3:**
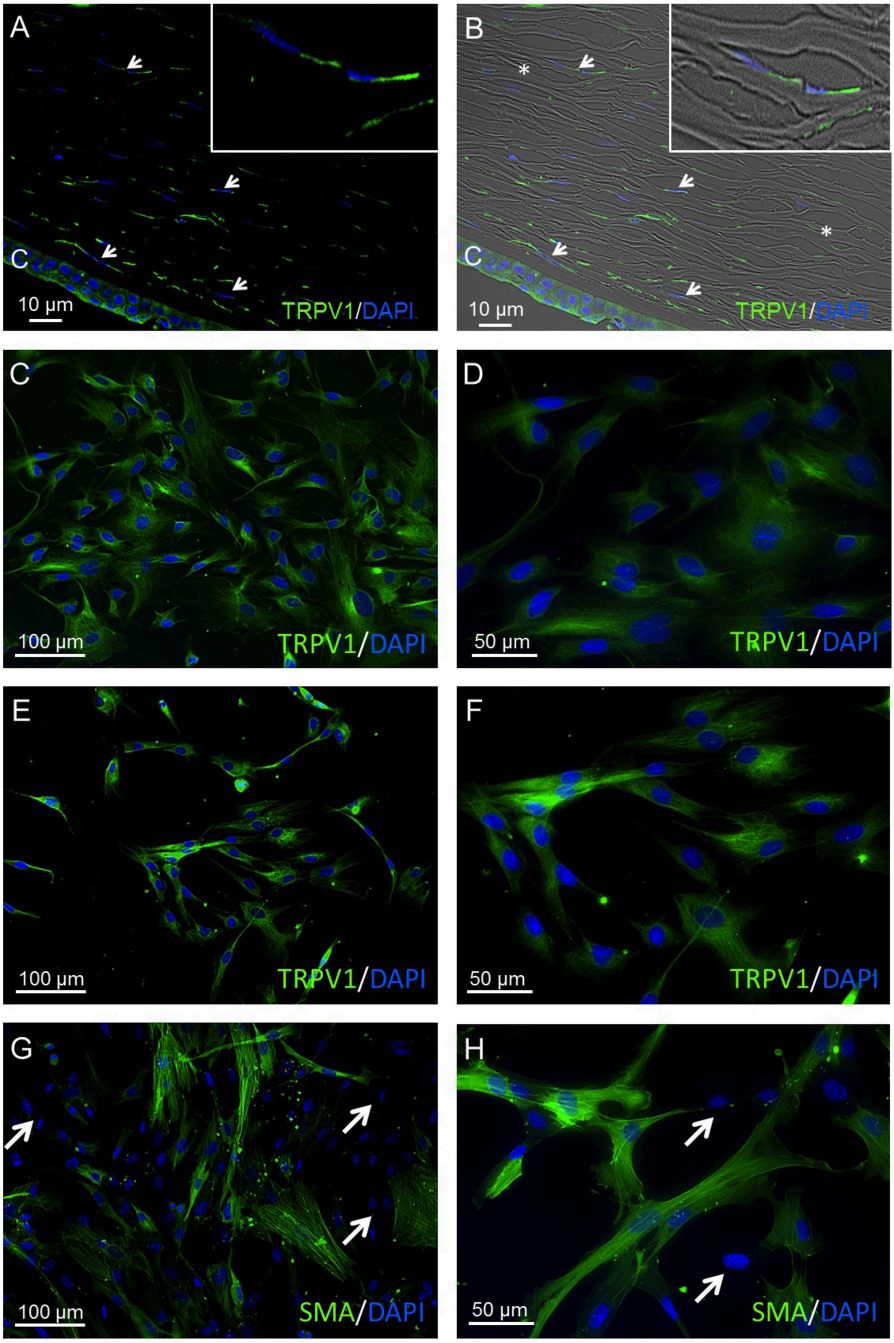
TRPV1 protein expression in human cornea and primary human cornea fibroblast (pHCF) cells. IF analyses show TRPV1 **(A,B)** in human cadaver corneal cross sections. Stromal keratocytes (arrows) and corneal epithelial cells (CEC) clearly have a distinct subcellular origin (green). Cell nuclei were counterstained with DAPI (blue). The overlay of fluorescence with bright field picture **(B)** demonstrates parallel arrangement of the collagen fibrils (^*^) and the stromal keratocytes in between the lamellae. Inlays show higher magnification of stromal keratocytes. Pictures are representative of IF staining patterns from five cadaver corneas (*n* = 5). IF analyses reveal TRPV1 expression in pHCF at passage 1 **(C,D)** and passage 5 **(E,F)**. **(G,H)** Anti-α-SMA antibody IF staining in primary human cornea fibroblast cells (passage 1). Arrows identify cells lacking IF staining signal. Notable, the green α-SMA IF staining pattern is partial and intense.

**Figure 4:**
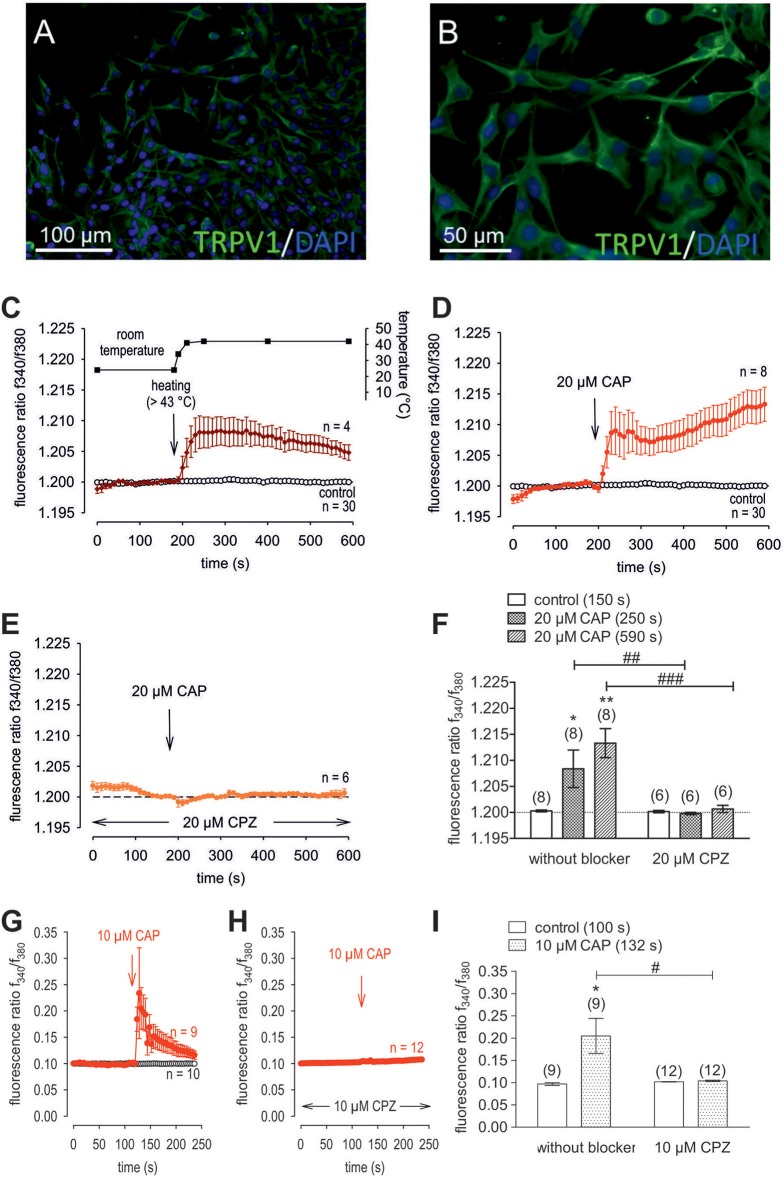
Confirmation of protein and functional TRPV1 expression in cultivated human corneal keratocytes (HCK). **(A,B)** Localization of TRPV1 in SV40-immortalized human corneal keratocytes (HCK). IF analysis reveal subcellular TRPV1 expression (green) in all HCK cells. Cell nuclei were counterstained with DAPI (blue) and pictures were merged. Pictures are representative of IF results from different cell passages. **(C)** Temperature increase from ≈ 23°C to > 43°C increased [Ca^2+^]_i_ (*n* = 4). The corresponding temperature time course is shown above the Ca^2+^ traces. The thermal and pharmacological changes were carried out at the time points indicated by arrows. **(D)** CAP (20 μM) induced an irreversible increase in Ca^2+^ influx (*n* = 8) whereas non-treated control cells maintained a constant Ca^2+^ baseline (*n* = 30). **(E)** Same experiment as shown in **(D)**, but in the presence of capsazepine (CPZ). CPZ (20 μM) suppressed the CAP-induced Ca^2+^ increase (*n* = 6). **(F)** Summary of the experiments with CAP and heat stimulation. The asterisks (^*^) designate significant increases in [Ca^2+^]_i_ with CAP (*n* = 8; *p* < 0.05 at the minimum; paired tested). The hashtags (#) indicate statistically significant differences in fluorescence ratios between CAP with and without CPZ (*n* = 6–8; *p* < 0.01 at the minimum; non-paired tested). **(G)** CAP (10 μM) induced a reversible increase in Ca^2+^ influx (*n* = 9) whereas non-treated control cells maintained a constant Ca^2+^ baseline (*n* = 10). **(H)** Same experiment as shown in **(G)**, but in the presence of capsazepine (CPZ). CPZ (10 μM) suppressed the CAP-induced Ca^2+^ increase (*n* = 12). **(I)** Summary of the experiments with CAP and CPZ. The asterisks (^*^) designate significant increases in [Ca^2+^]_i_ with CAP (*n* = 9; *p* < 0.05; paired tested). The hashtag (#) denotes a statistically significant difference in fluorescence ratios between CAP with and without CPZ (*n* = 9–12; *p* < 0.05; non-paired tested).

TRPV1 was identified in corneal stromal cells of human corneal cross sections (HCCS) as well as in primary human corneal fibroblast (pHCF) cells at different cell passages. The anti-TRPV1 antibody exhibited a consistent cytoplasmic staining pattern in all analyzed cell passages 1–5 (Figures 3A,F). Furthermore, α-SMA-derived green fluorescence was localized in the cytoplasm in about 50% of pHCF (passage 1) (Figures 3G,H). Some HCF cells were not stainable with any of the aforementioned antibodies (arrows), which is consistent with the presence of fibroblasts and myofibroblasts derived from keratocytes. Figures 4A,B demonstrate the localization of TRPV1 in SV40-immortalized HCK cells, which were used in the following functional assays.

### Functional TRPV1 channel expression

**Figure 5:**
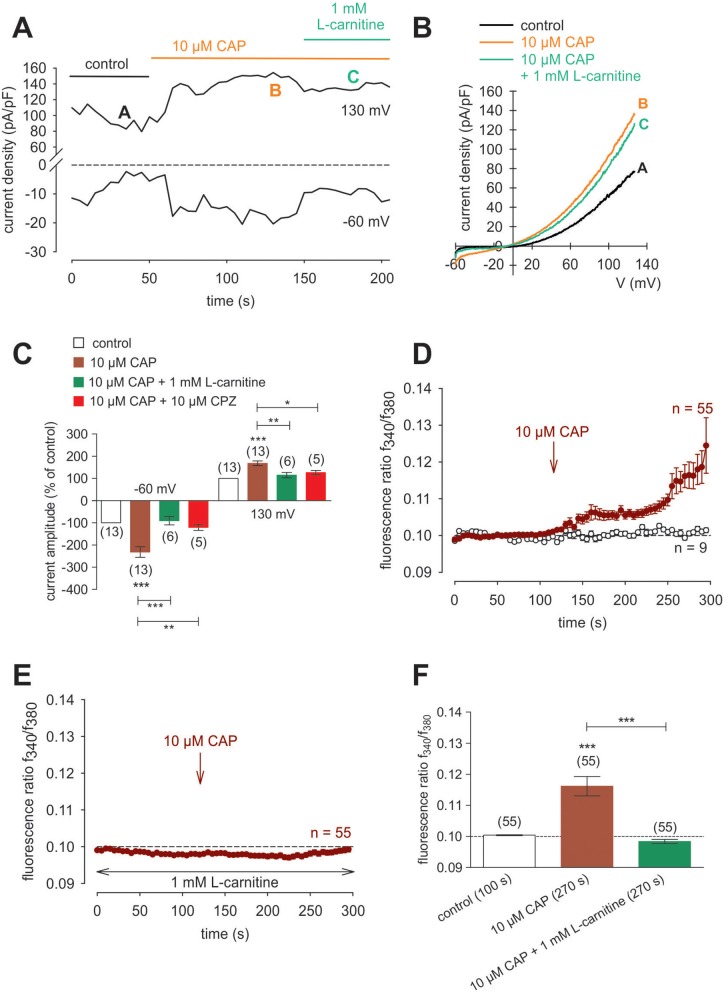
CAP activated whole-cell currents in HCK. **(A)** Time course recording of the current increases induced by CAP (10 μM) and decline after application of 1 mM L-carnitine. **(B)** Original traces of CAP-induced current responses to voltage ramps. Current densities are shown before application (labeled as A), during application of 10 μM CAP (labeled as B), and after addition of 1 mM L-carnitine (labeled as C). Calculated current densities obtained by normalizing currents to membrane capacitance as function of imposed voltage were derived from the traces shown in **(A)**. **(C)** Summary of the experiments with CAP, L-carnitine and CPZ. Ten micromolar CAP induced an increase of normalized maximal in- and outward whole-cell currents (for −60 mV control was set to −100%; for 130 mV control was set to +100%; *n* = 13). This increase could be suppressed by 1 mM L-carnitine and 10 μM CPZ, respectively (*n* = 5–6). Data passed the normality test and were statistically analyzed using one-way ANOVA (one-way analysis of variance). Selected pairs of columns were tested using Bonferroni post-test. The asterisks (^*^) designate significance differences between the columns (*n* = 5–13; *p* < 0.05 at the minimum). Significance level alpha = 0.05 (95% confidence intervals). **(D)** CAP (10 μM) induced an irreversible increase of intracellular Ca^2+^ concentration (n = 55) whereas non-treated control cells maintained a constant Ca^2+^ baseline (*n* = 9). **(E)** Same experiment as shown in **(D)**, but in the presence of L-carnitine. L-carnitine (1 mM) suppressed the CAP-induced Ca^2+^ increase (*n* = 55). **(F)** Summary of the experiments with CAP and L-carnitine. Data were statistically analyzed using one-way ANOVA (and Nonparametric) (one-way analysis of variance) (Kruskal-Wallis test). Selected pairs of columns were tested using Dunns post-test. The asterisks (^*^) designate significance differences between the columns (*n* = 55; *p* < 0.005). Significance level alpha = 0.05 (95% confidence intervals).

Raising the bath solution temperature to >43°C (Figure 4C, upper trace) increased the fluorescence ratio from 1.2000 ± 0.0001 to 1.2050 ± 0.0013 after 590 s in immortalized HCK (*n* = 4; Figure 4C). Similarly, activation of TRPV1 by 20 μM μM capsaicin (CAP) irreversibly increased the fluorescence ratio from 1.2000 ± 0.0002 to 1.213 ± 0.0028 after 590 s (*n* = 8; *p* < 0.01; Figure 4D), whereas this rise was blocked by 20 μM capsazepine (CPZ) (1.201 ± 0.0007; *n* = 6; *p* < 0.001; Figures 4E,F). To avoid desensitization and cell death with over stimulation, experiments were repeated using a lower concentration of CAP and CPZ (both 10 μM) in combination with an alternative fluorescence signal measuring setup (Figures 4G,H,I). The results are comparable. However, a recovery of the CAP-induced Ca^2+^ increase could be clearly suppressed in the presence of 10 μM CPZ (Figure 4H). More specifically, activation of TRPV1 by 10 μM CAP reversibly increased the fluorescence ratio from 0.0969 ± 0.0031 to 0.2050 ± 0.0394 after 132 s (*n* = 9; *p* < 0.05; Figure 4G), whereas this rise could be blocked by 10 μM CPZ (0.1041 ± 0,0017; *n* = 12; *p* < 0.05; Figures 4H,I). The changes and the underlying whole-cell currents were determined by measuring the time dependent changes in currents along with evaluating the plots of the corresponding current voltage relationships at the indicated time points: A, B and C (Figures 5A,B). At positive pipette potentials, CAP (10 μM) activated outwardly rectifying currents that were larger than those at negative voltages (Figure 5B). As already observed in HCjEC, L-carnitine (1 mM) is a TRPV1 antagonist (15) and it reduced maximal negative and maximal positive current amplitudes induced by a voltage step from −60 to +130 mV (% of control) (15) shown in Figure 5C. In each measured cell, 10 μM CAP increased both in- and outward currents, whereas 1 mM L-carnitine suppressed them (*n* = 6; *p* < 0.05 at the minimum). In addition, functional TRPV1 expression could be also documented with the classical TRPV1 antagonist CPZ (10 μM) (*n* = 5; *p* < 0.05; Figure 5C). In some previous studies as well as this study, 20 μM CAP was used to elicit physiological responses in human corneal endothelial cells (11), corneal epithelial cells (60), retinoblastoma cells (70) and in neuroendocrine tumor cells (71). Regarding the effect of L-carnitine, fluorescence Ca^2+^ imaging results mirrored those obtained in the patch-clamp experiments (Figures 5D,F). The CAP-induced Ca^2+^ increase (*n* = 55; control *n* = 9) could be clearly suppressed in the presence of 1 mM L-carnitine (Figures 5E,F; *n* = 55; *p* < 0.005). Overall, functional TRPV1 channel expression in HCK was confirmed using different CAP and CPZ concentrations. This was evident because the results obtained with two different fluorescence calcium monitoring systems corresponded with those detected with the patch-clamp technique. In summary, functional TRPV1 expression is present in all types of stromal cells.

### TRPM8 gene and protein expression

**Figure 6:**
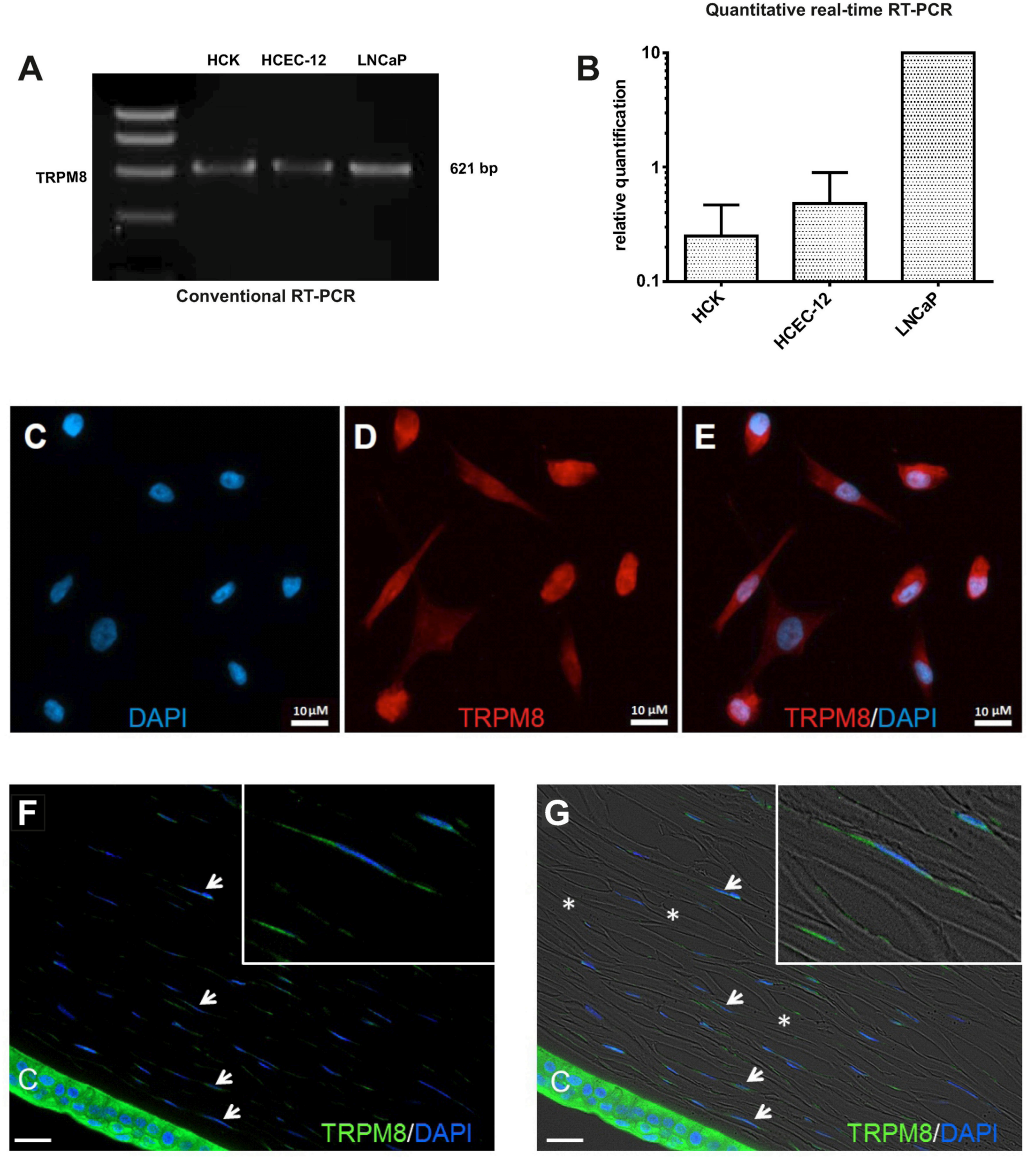
TRPM8 gene and protein expression in HCK. **(A)** Conventional RT-PCR indicates mRNA signal of TRPM8 amplicon (621-bp) in human corneal keratocytes (HCK), human corneal endothelial cells (HCEC-12) and human prostate cancer cells (LNCaP) as a positive control. **(B)** Quantitative real-time RT-PCR analysis. The data were normalized to LNCaP with the positive mRNA signal. GAPDH was used as a housekeeping gene for normalization. Data are the mean ± SEM of 3 independent triplicate experiments. **(C–E)** Immunocytochemistry documents subcellular TRPM8 localization. **(C)** Nuclear staining with DAPI (blue). **(D)** TRPM8 positive cells (red). **(E)** merged. **(F,G)** IF analyses identify TRPM8 expression in human cadaver corneal cross sections. Stromal keratocytes (arrows) and corneal epithelial cells (CEC) clearly show a subcellular IF signal (green). Cell nuclei were counterstained with DAPI (blue). The overlay of fluorescence with bright field picture demonstrate parallel arrangement of the collagen fibrils (^*^) and stromal keratocytes interspersed between the lamellae. Inlays show higher magnification of stromal keratocytes. Pictures are representative of IF results obtained from five cadaver corneas (*n* = 5).

RT-PCR and semi-quantitative real-time PCR identified TRPM8 gene expression in HCK (Figures 6A,B) based on generating the predicted TRPM8 621-bp amplicon (Figure 6A). qPCR confirmed its identity because the size of this product was identical with its positive control in the LNCaP cell line (72, 73) and corneal endothelial cells (41). In addition, TRPM8 immunostaining expression was identified in both the cell membrane and peri-nuclear regions (Figures 6C,D,E). Absence of immunostaining caused by omission of the primary antibody excluded nonspecific secondary antibody staining. IF analyses also detected TRPM8 expression, on keratocytes in HCCS (Figures 6F,G).

### Functional TRPM8 channel expression

**Figure 7:**
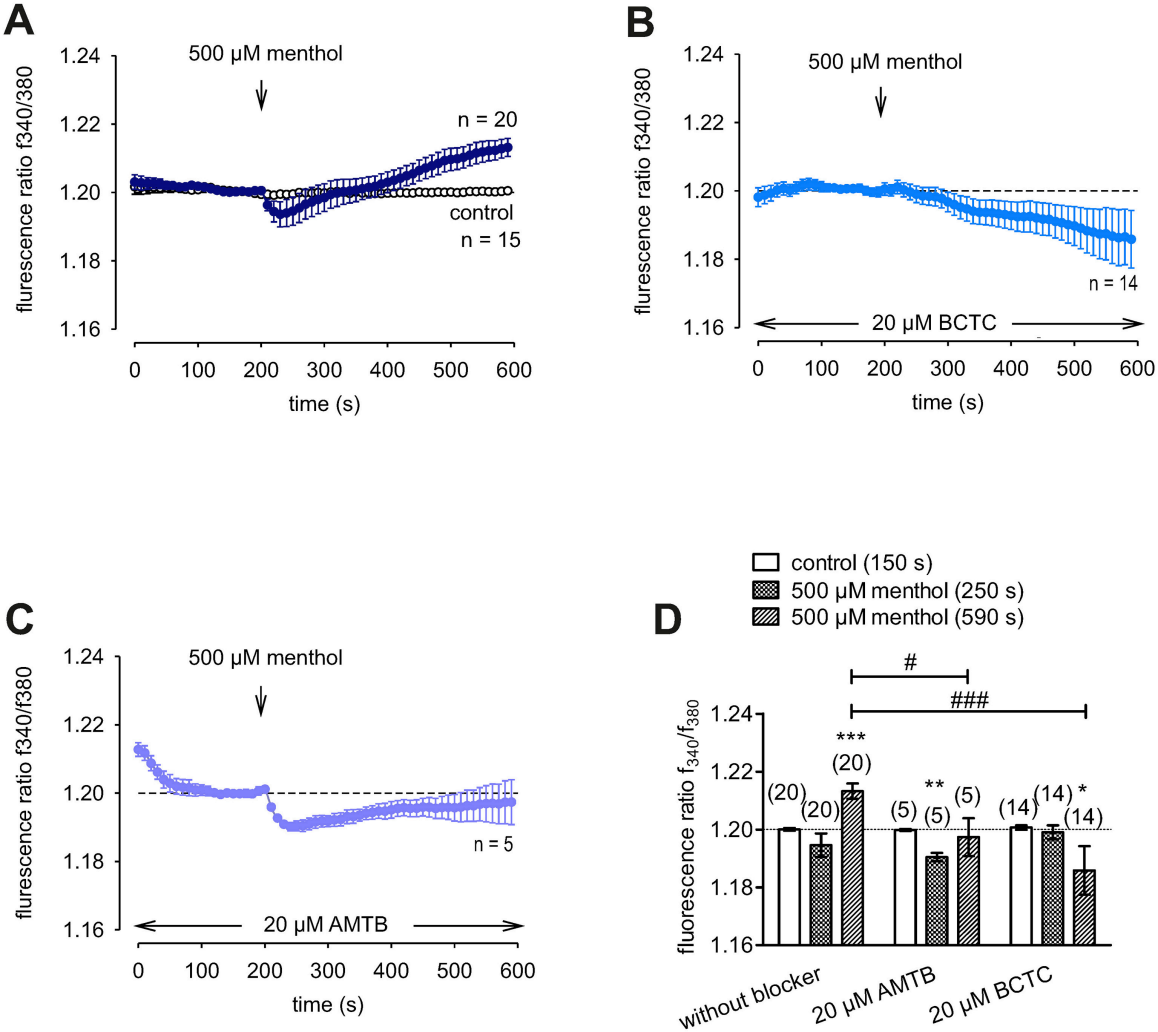
Confirmation of functional TRPM8 expression in HCK. The pharmacological changes were carried out at the time points indicated by arrows. Data are mean ± SEM of 5-20 experiments. **(A)** Menthol (500 μM) induced an irreversible increase in Ca^2+^ influx (*n* = 20) whereas non-treated control cells showed a constant Ca^2+^ baseline (*n* = 15). **(B,C)** Same experiments as shown in **(A)**, but in the presence of BCTC **(B)**, and AMTB **(C)**. Both AMTB and BCTC (both 20 μM) clearly suppressed the menthol-induced Ca^2+^ increase (*n* = 5–14). **(D)** Summary of the experiments with menthol, AMTB, and BCTC. The asterisks (^*^) designate significance difference between with and without menthol, a TRPM8 agonist (*n* = 5–20; *p* < 0.05 at the minimum; paired tested). The hashtags (#) indicate statistically significant differences of fluorescence ratios with and without BCTC or AMTB (*p* < 0.05 at the minimum; unpaired tested).

**Figure 8:**
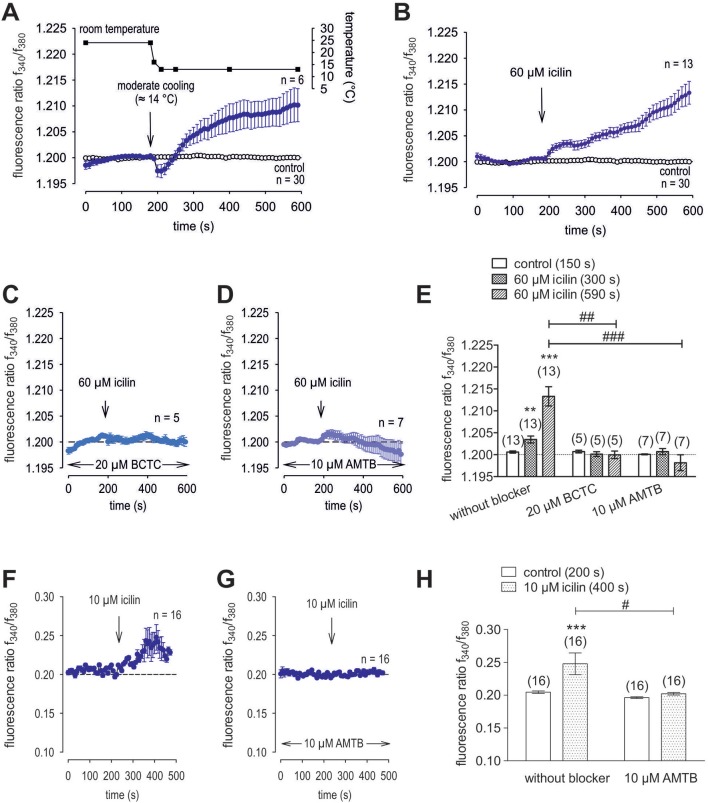
Additional confirmation of functional TRPM8 expression in HCK. The thermal and pharmacological changes were carried out at the time points indicated by arrows. Data are mean ± SEM of 5-30 experiments. **(A)** Temperature lowering from ≈ 23°C to ≈ 14°C resulted in an increase [Ca^2+^]_i_ influx (*n* = 6). The corresponding temperature time course is shown above the Ca^2+^ traces. Untreated cells maintained stable [Ca^2+^]_i_ levels (control; *n* = 30). **(B)** Icilin (60 μM) induced an irreversible increase in Ca^2+^ influx (*n* = 13) whereas non-treated control cells showed a constant Ca^2+^ baseline (*n* = 30). **(C)** Same experiment as shown in **(B)**, but in the presence of BCTC. BCTC (20 μM) clearly suppressed the icilin-induced Ca^2+^ increase (*n* = 5). **(D)** Same experiment as shown in **(B)**, but in the presence of AMTB with the same result as obtained with BCTC (*n* = 7). **(E)** Summary of the experiments with icilin, BCTC and AMTB. The asterisks (^*^) indicate significant differences between [Ca^2+^]_i_ levels with icilin and in the presence or absence of BCTC or AMTB (*n* = 5–13; *p* < 0.01 at the minimum; paired tested). The hashtag (#) denotes a statistically significant difference in the fluorescence ratios between icilin with and without BCTC and AMTB, respectively (*n* = 5–13; *p* < 0.01 at the minimum; unpaired tested). **(F)** Icilin (10 μM) induced a partial reversible increase in Ca^2+^ influx (*n* = 16). **(G)** Same experiment as shown in **(F)**, but in the presence of AMTB. AMTB (10 μM) suppressed the icilin-induced Ca^2+^ increase (*n* = 16). **(H)** Summary of the experiments with icilin. The asterisks (^*^) designate significant increases in [Ca^2+^]_i_ with icilin (*n* = 16; *p* < 0.005; paired tested). The hashtag (#) denotes a statistically significant difference in fluorescence ratios between icilin with and without AMTB (*n* = 16; *p* < 0.05; non-paired tested).

Menthol (200 μM−1 mM) probed for functional TRPM8 expression (74). This selective TRPM8 agonist at 500 μM irreversibly increased the f_340nm_/f_380nm_ ratio from 1.2000 ± 0.0004 to 1.213 ± 0.003 after 590 s in HCK (*n* = 20; p = 0.001; Figure 7A), whereas the mixed TRPV1/ TRPM8 antagonist, 20 μM BCTC, blocked the menthol-induced Ca^2+^ increase (1.186 ± 0.0084; *n* = 14; *p* < 0.001); Figure 7B) (22). Similarly, 20 μM AMTB, a selective TRPM8 antagonist (21, 75), completely blocked this response (1.197 ± 0.0066; *n* = 5; p < 0.05; Figure 7C). Furthermore, TRPM8 involvement is indicated because constant moderate cooling (≈14°C; Figure 8A, upper trace) increased the f_340nm_/f_380nm_ ratio from 1.2000 ± 0.0001 to 1.2100 ± 0.0032 after 590 s (*n* = 6; Figure 8A) whereas in non-stimulated controls, the f_340nm_/f_380nm_ ratio was invariant (*n* = 30; Figure 8A). Icilin, is a super-cooling agent that has higher potency and efficacy than menthol in cellular and behavioral studies (44, 76). Sixty-μM icilin irreversibly increased the fluorescence ratio from 1.2010 ± 0.0003 to 1.2130 ± 0.022 after 590 s (*n* = 13; *p* < 0.001; Figure 8B), which BCTC (20 μM) (Figure 8C) and AMTB blocked (10 μM; 1.1980 ± 0.0018; *n* = 7; *p* < 0.001; Fig 8D). The statistical evaluation is shown in Figure 8E. Similar results were obtained with a lower icilin concentration (10 μM) in combination with AMTB (Figures 8F,G,H). Even though both of the increases in Ca^2+^ induced by menthol and icilin were blocked by AMTB, the difference in their increases stems from the fact that each of these mixed TRPM8/TRPA1 agonists have different TRPM8 selectivity (77). Although high icilin concentrations were used in human corneal epithelial cells [e.g., 15–60 μM (42)], 10 μM instead of 60 μM icilin was used in the aforementioned set of experiments to avoid any confounding effects with overstimulation. As shown in Figure 8F, a partial reversible calcium response could be observed in comparison to the irreversible calcium response shown in Figure 8B of the same experiment. Since icilin activates both TRPM8 and TRPA1 (78, 79), AMTB (10 μM), a highly selective TRPM8 blocker, was used to probe for TRPM8 (21, 80). Specifically, icilin reversibly increased the fluorescence ratio from 0.2045 ± 0.0019 to 0.2477 ± 0.0165 after 400 s (*n* = 16; *p* < 0.001; Figure 8F), which AMTB blocked (10 μM; 0.2021 ± 0.0020; *n* = 16; *p* < 0.001; Figures 8G,H). In summary, functional TRPM8 channel expression in HCK was confirmed using a lower icilin concentration in an alternative calcium imaging setup.

### Cooling compounds induce increases in TRPM8-mediated whole-cell currents

**Figure 9:**
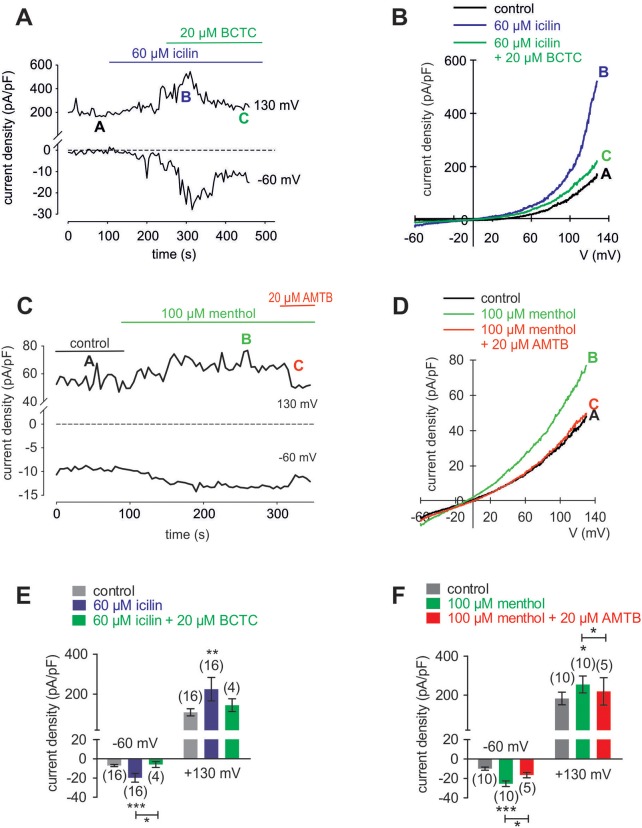
Menthol and icilin activated whole-cell currents in HCK. **(A)** Time course recording showing the current increases induced by icilin (60 μM) and declines after application of 20 μM BCTC. **(B)** Original traces of icilin-induced current responses to voltage ramps. Current densities are shown before application (labeled as A), during application of 60 μM icilin (labeled as B), and after addition of 20 μM BCTC (labeled as C). Current densities as function of voltage were derived from the traces shown in **(A)**. **(C)** Time course recording showing the current increases induced by menthol (100 μM) and declines after application of 20 μM AMTB. **(D)** Original traces of menthol-induced current responses to voltage ramps. Current densities are shown before application (labeled as A), during application of 100 μM menthol (labeled as B), and after addition of 20 μM AMTB (labeled as C). Current densities as function of voltage were derived from the traces shown in **(C)**. **(E)** Summary of patch-clamp experiments with icilin and BCTC. The asterisks (^*^) indicate statistically significant differences between in- and outward whole-cell currents with and without icilin (*n* = 16; *p* < 0.001; paired tested) and significant difference of inward currents between icilin with and without BCTC (*n* = 4; *p* < 0.05; unpaired tested). **(F)** Summary of patch-clamp experiments with menthol and AMTB. The asterisks (^*^) indicate statistically significant differences of in- and outward whole-cell currents with and without menthol (*n* = 10; *p* < 0.001; paired tested) and significant difference of inward currents between menthol with and without AMTB (*n* = 5; *p* < 0.05; paired tested).

In HCK, the increases in inward currents induced by 60 μM icilin (Figures 9A,B) are mainly attributable to Ca^2+^ influx driven by the favorable electrochemical gradient established by a large chemical driving force and a negative intracellular membrane voltage whereas the Na^+^ electrochemical gradient is at lower levels since the internal solution is not Na^+^-free. At −60 mV, the inward currents rose from −5.4 ± 1.1 (control) to −18.4 ± 4.7 pA/pF (*p* < 0.001; *n* = 16; Figure 9E), which is attributable to increasing the inwardly directed electrical driving force whereas the control currents remained at lower levels (Figure 9A). BCTC suppressed this rise to −6.0 ± 3.0 pA/pF (*p* < 0.05; *n* = 4; Figure 9E). Similarly, menthol (100 μM) increased the inward currents from −9.9 ± 1.6 pA/pF (control) to −25.4 ± 2.9 pA/pF (*p* < 0.01; *n* = 10; and 20 μM AMTB suppressed this rise to −16.5 ± 2.8 pA/pF (*p* < 0.05; *n* = 5; Figures 9C,F). This result affirms cell membrane delimited functional TRPM8 expression.

### VEGF-induced increases in Ca^2+^ influx and whole-cell currents

**Figure 10:**
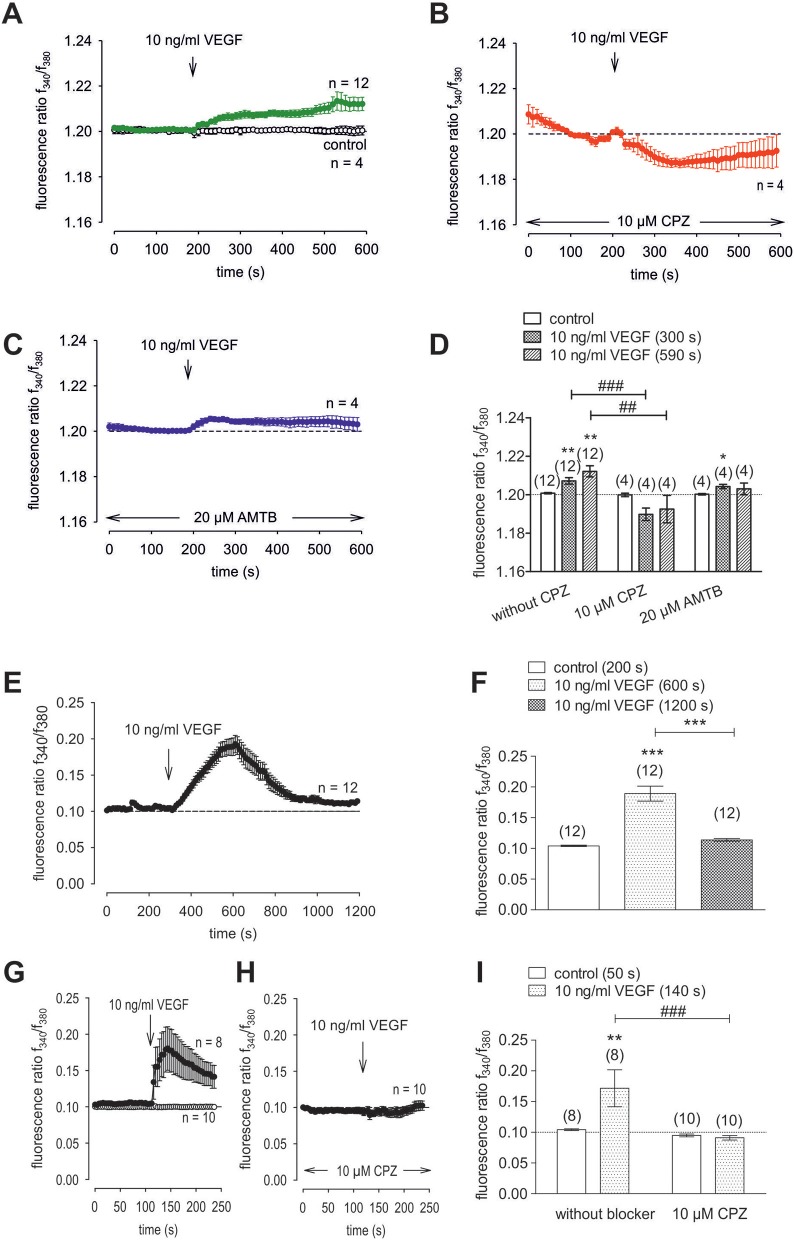
VEGF elicits increases in Ca^2+^ entry through TRPV1 activation in HCK. The pharmacological changes were carried out at the time points indicated by arrows. Data are mean ± SEM of 4-12 experiments. **(A)** VEGF (10 ng/ml) induced an irreversible Ca^2+^ influx (*n* = 12) whereas non-treated control cells showed a constant Ca^2+^ baseline (*n* = 4). **(B,C)** Same experiments as shown in **(A)**, but in the presence of CPZ (B) and AMTB (C). Whereas, CPZ (10 μM) clearly suppressed the VEGF-induced Ca^2+^ increase (*n* = 4) AMTB (20 μM) failed to suppress the VEGF-induced Ca^2+^ increase (*n* = 4). **(D)** Summary of the experiments with VEGF, CPZ and AMTB. The asterisks (^*^) designate significance differences with and without the respective TRPV1 and TRPM8 channel antagonists (CPZ/AMTB) (*n* = 4–12; *p* < 0.05 at the minimum; paired tested). The hashtags (#) indicate statistically significant differences of fluorescence ratios with and without CPZ (*n* = 4–12; *p* < 0.01 at the minimum; unpaired tested). **(E)** VEGF (10 ng/ml) induced a reversible Ca^2+^ increase (20 min) (*n* = 12). **(F)** Summary of the long-term recording with VEGF. The asterisks (^*^) designate significance differences with and without VEGF (*n* = 12; *p* < 0.001). **(G)** VEGF (10 ng/ml) induced a Ca^2+^ increase (*n* = 8) whereas non-treated control cells showed a constant Ca^2+^ baseline (*n* = 10). **(H)** Same experiments as shown in **(G)**, but in the presence of CPZ. CPZ (10 μM) clearly suppressed the VEGF-induced Ca^2+^ increase (*n* = 10). **(I)** Summary of the experiments with VEGF and CPZ. The asterisks (^*^) designate significance in [Ca^2+^]_i_ with VEGF (*n* = 8; *p* < 0.01; paired tested). The hashtag (#) denotes a statistically significant difference of fluorescence ratios with and without CPZ (*n* = 8–10; *p* < 0.001; unpaired tested).

**Figure 11:**
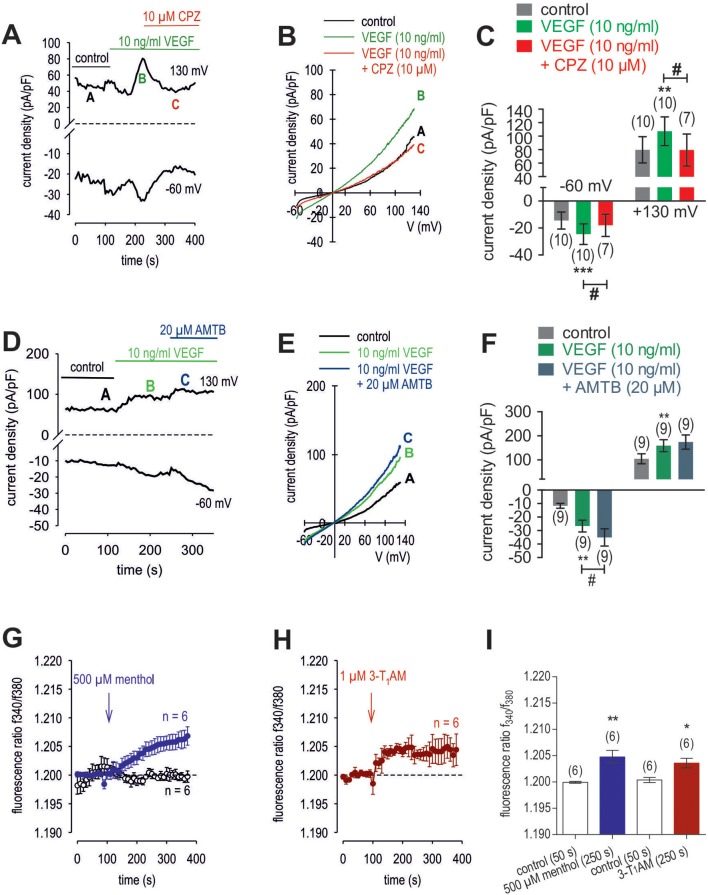
Effect of VEGF, menthol and 3-T_1_AM on Ca^2+^ regulation in HCK. **(A)** Time course recording showing the current increases induced by 10 ng/ml VEGF and current density levels after adding CPZ (10 μM). There was a delayed increase in the whole-cell currents. **(B)** Original traces of VEGF-induced current responses to voltage ramps. Current densities are shown before application (labeled as A), during application of VEGF (labeled as B), and after addition of CPZ (labeled as C). Current densities as function of voltage were derived from the traces shown in panel A. Notably, VEGF-induced in- and outward currents declined in the presence of CPZ. **(C)** Summary of the experiments with VEGF and CPZ. The asterisks (^*^) indicate statistically significant differences of VEGF-induced increases of in- and outward currents (*n* = 10; *p* < 0.01 at the minimum; paired tested). The hashtag (#) denotes a statistically significant difference in the whole-cell inward currents with and without CPZ (*n* = 7–10; *p* < 0.05; unpaired tested). **(D)** Time course recording showing the current increases induced by 10 ng/ml VEGF and current density levels after adding AMTB (20 μM). **(E)** Original traces of VEGF-induced current responses to voltage ramps. Current densities are shown before application (labeled as A), during application of VEGF (labeled as B), and after addition of AMTB (labeled as C). Current densities as function of voltage were derived from the traces shown in **(D)**. **(F)** Summary of the experiments with VEGF and AMTB. Same statistical analyses as those described in **(C)** but with AMTB instead of CPZ (*n* = 9; ^*^p < 0.01; ^#^*p* < 0.05). **(G)** Menthol (500 μM) induced a Ca^2+^ increase (*n* = 6) whereas non-treated control cells showed a constant Ca^2+^ baseline (*n* = 6). **(H)** Same experiments as shown in **(G)**, but with 3-T_1_AM (1 μM) (*n* = 6). **(I)** Summary of the experiments with menthol and 3-T_1_AM. The asterisks (^*^) designate significance in [Ca^2+^]_i_ with menthol (*n* = 6; ^**^p < 0.01) or with 3-T_1_AM (*n* = 6; ^*^p = 0.05; both paired tested).

To clarify if VEGFR mediates TRPV1 activation in HCK, the effects of 10 ng/ml VEGF on Ca^2+^ influx and whole-cell currents were measured with and without CPZ. This growth factor increased the f_340nm_/f_380nm_ ratio from 1.2010 ± 0.0003 to 1.2120 ± 0.0030 (590 s) (*n* = 12; *p* < 0.01, Figure 10A) whereas cells pretreated with 10 μM CPZ caused the Ca^2+^ transients to even fall below their baseline value (1.1930 ± 0.0070; *n* = 4; *p* < 0.01) (Figures 10B,D). To ascertain if VEGFR interacts also with TRPM8, we determined if 20 μM AMTB inhibited a VEGF induced Ca^2+^ transient. Unlike CPZ, AMTB had no effect on VEGF-induced increases in the f_340nm_/f_380nm_ ratio indicating that the increases in currents induced by VEGF are only due to crosstalk between VEGFR and TRPV1 (Figures 10C,D). Long-term calcium imaging recordings (20 min) revealed a recovery effect of VEGF (*n* = 12; *p* < 0.005) (Figures 10 E,F). The CPZ-induced suppression of the VEGF effect was confirmed using the aforementioned alternative fluorescence calcium imaging setup (*n* = 8–10; *p* < 0.005; unpaired tested) (Figures 10 G,I). Specifically, VEGF increased the f_340nm_/f_380nm_ ratio from 0.1041 ± 0.001 (200 s) to 0.1892 ± 0.012 (600 s) (recovery occurred to 0.1139 ± 0.002 (1,200 s) (*n* = 12; *p* < 0.01; paired tested; Figures 10E,F) whereas CPZ was again able to suppress the VEGF-induced Ca^2+^ increase. Without CPZ, the f_340nm_/f_380nm_ ratio increased from 0.1043 ± 0.001 (50 s) to 0.1717 ± 0.030 (140 s) (*n* = 8; *p* < 0.01; paired tested) whereas this increase was suppressed in the presence of 10 μM CPZ to 0.0911 ± 0.004 (140 s) (*n* = 10; *p* < 0.005; unpaired tested; Figures 10G,H,I). In addition, VEGF (10 ng/ml) increased the whole-cell inward currents from −14 ± 6 pA/pF to −25 ± 8 pA/pF (*n* = 10; *p* < 0.001), whereas the outward currents increased from 80 ± 19 pA/pF to 107 ± 21 pA/pF (*n* = 10; *p* < 0.01) (Figures 11A,C). Consistent with crosstalk between VEGFR and TRPV1, these current rises were delayed in some of the experiments (Figure 11A). In contrast, 10 μM CPZ immediately inhibited the rises in the inward currents to −18 ± 8 pA/pF; *n* = 7; *p* < 0.05) and the outward currents to 79 ± 24 pA/pF (*n* = 7; *p* < 0.05; Figures 11A,B,C). On the other hand, 10 ng/ml VEGF increased outward currents from 105 ± 21 pA/pF to 159 ± 25 pA/pF (*n* = 9; *p* < 0.01) and inward currents increased from −12 ± 2 pA/pF to −27 ± 4 pA/pF (*n* = 9; *p* < 0.01; Figures 11D,E,F). AMTB did not suppress these currents (Figure 11D). This antagonist even slightly increased the inward currents to −35 ± 6 pA/pF (*n* = 9; *p* < 0.05). To confirm that VEGFR-mediates TRPV1 activation, CPZ preincubation blocked VEGF-induced Ca^2+^ transients using the aforementioned alternative fluorescence detection method. Taken together, VEGFR activation by VEGF solely increases Ca^2+^ influx through increasing TRPV1-induced Ca^2+^ influx and its underlying whole-cell currents.

### Thyronamine (3-T_1_AM) suppresses VEGF-induced increases in whole-cell currents

**Figure 12:**
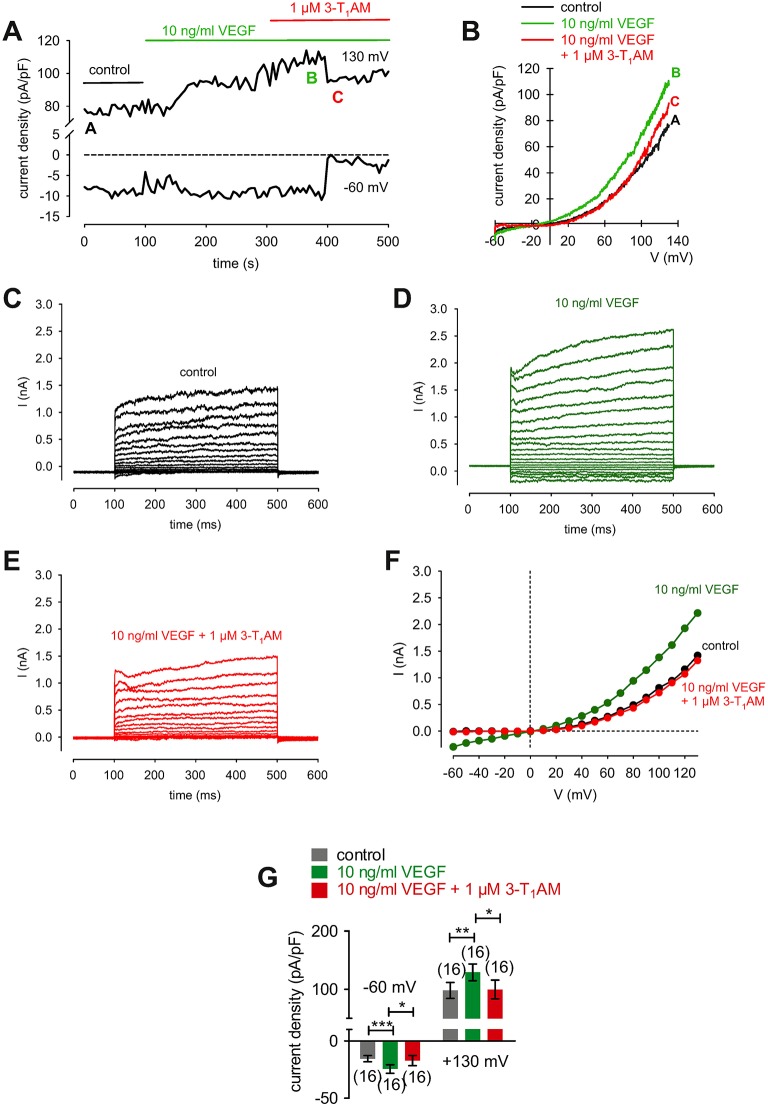
3-T_1_AM suppresses VEGF-induced increases in whole-cell currents in HCK. **(A)** Time course recording showing the current increases induced by 10 ng/ml VEGF and current density levels after adding 3-T_1_AM (1 μM). There was a delayed increase and decrease of whole-cell currents, respectively. **(B)** Original traces of VEGF-induced current responses to voltage ramps. Current densities are shown before application (black labeled as A), during application of VEGF (green labeled as B), and after addition of 3T_1_AM (red labeled as C). Current densities as function of voltage were derived from the traces shown in **(A)**. Notably, there was a decrease of VEGF-induced in- and outward currents in the presence of 3-T_1_AM. **(C)** Whole-cell currents under control conditions induced by depolarization from −60 to 130 mV in 10 mV steps (400 ms). **(D)** Increased whole-cell currents (green traces) in the presence of 10 ng/ml VEGF. **(E)** Decreased whole-cell currents (red traces) in the additional presence of 1 μM 3-T_1_AM. **(F)** Effect of VEGF and 3-T_1_AM summarized in a current/voltage plot [I-V plot, data obtained from **(C–E)**]. The black trace (filled circles) was obtained under control conditions. The green trace was obtained in the presence of 10 ng/ml VEGF and the lower red trace in the additional presence of 3-T_1_AM. An inhibitory effect could be observed. **(G)** Summary of the experiments with VEGF and 3-T_1_AM. The asterisks (^*^) indicate statistically significant differences of in- and outward currents with and without 3-T_1_AM (*n* = 16; *p* < 0.05 at the minimum; paired tested).

3-T_1_AM (1 μM) increased the f_340nm_/f_380nm_ ratio from 1.2000 ± 0.0005 to 1.2040 ± 0.0009 (350 s) in HCK (*n* = 6; *p* < 0.05) (Figure 11H), which was similar to the increase induced by menthol (1.2050 ± 0.0012; *n* = 6; *p* < 0.01) (Figures 11G, I). The increases in the inward whole-cell currents induced by VEGF (10 ng/ml) shown in Figure 11 are similar to those shown in Figure 12. Figure 12A shows maximum in- and outward whole-cell currents under control conditions (marked as A) and in the presence of 10 ng/ml VEGF (shown in green and marked as B). After extracellular application of 1 μM 3-T_1_AM, the currents decreased after a short delay (shown in red and marked as C). The corresponding current voltage relationships at the aforementioned points of time (A, B, and C) are shown in **Figure** 1**2B**. This experiment was repeated using a step protocol instead of a voltage ramp protocol (from −60 to +130 mV in 10 mV steps at a holding potential of 0 mV). Figure 12C shows the corresponding whole-cell currents under control conditions, which were increased in the presence of VEGF (Figure 12D). Notably, the inward currents were completely suppressed in the presence of 3-T_1_AM since the inward currents are mainly attributable to increases in Ca^2+^ influx (Ca^2+^ free internal solution) (Figure 12E). The corresponding current voltage relationships from the recordings shown in Figures 12C,D,E are summarized in Figure 12F, which clearly demonstrate that the VEGF-induced current increase could be blocked by 3-T_1_AM. Figure 12G summarizes the statistical analysis of all patch-clamp experiments. Specifically, the inward currents rose from −15 ± 3 pA/pF to −24 ± 4 pA/pF (*n* = 16; *p* < 0.0005). Similar to CPZ, 1 μM 3-T_1_AM suppressed the inward currents (−19 ± 5 pA/pF; *n* = 16; *p* < 0.05; Figure 12G). The same pattern was seen in the outward currents. VEGF increased the mean outward currents from 98 ± 14 pA/pF to 129 ± 14 pA/pF (*n* = 16; *p* < 0.01), which were suppressed to 100 ± 16 pA/pF by 3-T_1_AM (*n* = 16; *p* < 0.05; Figure 12G). Taken together, 3-T_1_AM suppressed VEGF-induced increases in the whole-cell currents.

## Discussion

### Limitations of therapeutic intervention of corneal neovascularization

Corneal neovascularization (CNV) is a severe pathological condition that may profoundly impair vision. Although its clinical impact is very well known, novel strategies are still needed to prevent infiltration of incompletely formed blood vessels. This maladaptive response to either infection, trauma, toxic agents or inflammation accounts for a significant number of patients seen in clinical practice (81). Angiogenesis is also a significant risk factor for keratoplasty because neovascularization develops in 41% of the cases undergoing this procedure (82). Furthermore, medical interventions are only partially successful because they do not fully reduce neovascularization through topical, subconjunctival, and intraocular application of bevacizumab [reviewed (83)]. Alternatively, glucocorticoids are used but they do not regress preexisting CNV [reviewed (84)]. Furthermore, chronic usage of steroids to reduce inflammation often has ocular side effects such as cataracts, glaucoma and superinfection (85). Another limitation involves their short duration of action. Rapamycin also has limited effectiveness in inhibiting CNV induced by a chemical burn through suppressing the *mammalian target of rapamycin* (mTOR) signaling pathway in a mouse corneal wound healing model (86, 87). Even though CNV declined, it was accompanied by increases in proinflammatory cytokines and decreases in anti-inflammatory cytokines. These latter effects may instead aggravate infection driven immune-induced inflammation (88). Such complications account for continuing efforts to develop alternative strategies for more effective suppression of neovascularization (89).

### Biomarker validation of HCK identity

Biomarker expression patterns confirmed the practicality of using the HCK cell line as an *in vitro* test model (Figure 1). Even though, LUM is also expressed by other cell types in the heart and lung (32, 90), it is coexpressed with KTN, which is a more selective keratocyte biomarker than LUM (32) (Figure 1C). Furthermore, CD90, a mesenchymal stem cell marker, was evident (Figure 2). This expression pattern documents the heterogeneity of cell types found in stromal isolates of human corneal tissue (25, 26, 91). Myofibroblasts were also detected based on a more intense α-SMA staining pattern than that of KTN (Figure 1B). Furthermore, fibroblast and myofibroblast coexistence is consistent with numerous reports showing that keratocyte phenotype maintenance is very condition dependent. For example, environmental stress or tissue culture medium content changes can cause keratocytes to undergo a differentiation program. As a result, this transition can lead to fibroblasts and ultimately myofibroblast differentiation (66, 68).

### Specificity of TRPV1 activation

TRPV1 protein expression in stromal keratocytes, HCCS, pHCF (Figure 3) and HCK (Figures 4A,B), agrees with its presence in human corneal fibroblasts (13). The CAP-induced Ca^2+^ transients in corneal epithelial cells closely resemble those obtained with the HCK cell line (16). The irreversibility of these transients in some tissues may be related to differences in its tissue specific binding to TRPV1. Functional TRPV1 expression was also confirmed by showing that raising the bath temperature above 43°C induced rises in Ca^2+^ levels that were partially reversible (92). Differences in reversibility were also found between pterygial cells and HCjEC. The poor reversibility in the immortalized HCK cell line corresponds with what was described in non-malignant pterygial cells and healthy HCjEC (16). As shown in Figure 3, not all HCK cells were stainable. This staining inhomogeneity is consistent with the presence of fibroblasts and myofibroblasts derived from keratocytes. Notably, TRPV1 and α-SMA are coexpressed, which is characteristic of myofibroblasts (38). Furthermore, their coexpression was described in a report showing that TRPV1 upregulation promotes α-SMA expression during myofibroblast transdifferentiation (93). Such an association may be needed for TRPV1 to mediate control of actin assembly and disassembly that occurs during upregulation of this contractile protein. This type of dependence was described in F11 cells transduced with retroviral particles obtained from a triple transfection of human embryonic kidney (HEK)293T cells with TRPV1 plasmids (93). CAP concentrations (10–20 μM) led to Ca^2+^ transients and whole-cell current increases (Figures 4D,I, 5A,D). These effects are in line with those reported in studies using human corneal endothelial cells (11), corneal epithelial cells (60), retinoblastoma cells (70), and in neuroendocrine tumor cells (71). The CAP-induced changes of ≈20 pA/pF in either direction are moderate (e.g., Figures 5A,B,C) and may be mixed by heterogeneous responses of different cell types in the HCK cell line. As previously described, L-carnitine is able to suppress TRPV1 activation (Figures 5A,B,C) and Ca^2+^ influx (Figures 5D,E,F), which makes this osmoprotectant relevant for possible use in a clinical setting (e.g., dry eye) (15, 94–96). Taken together, CAP has adequate TRPV1 selectivity to be used as a marker of its expression (46, 24). Even though CPZ only blocks the vanilloid site on TRPV1, it is the first identified competitive antagonist of capsaicin, which is a sensory neuronal excitant. Accordingly, it is universally accepted as a relevant TRPV1 probe (24, 46, 97).

### Specificity of TRPM8 activation

TRPM8 gene and protein expression levels (Figures 6A,B,C,D,E) in HCK and HCCS cells (Figures 6F,G) are similar to those described in human corneal (HCEC) and conjunctival epithelial cells (HCjEC) (42, 43). Even though there is a poor correspondence between the extensive and rather intense TRPM8 immunostaining and low level of TRPM8 gene expression, this disparity may be attributable to reported limited selectivity of the commercially available TRPM8 antibodies (16, 42, 43). Nevertheless, menthol (100–500 μM) induced Ca^2+^ transients and increases in whole-cell currents corresponding to those reported in HCEC and HCjEC (42, 43). These high menthol concentrations were appropriate because concentrations as high as 1 mM were used for describing functional TRPM8 expression in some other studies (74). Similarly, inhibition by AMTB of these menthol induced responses confirmed TRPM8 involvement (Figure 7A) (98). Non-specific menthol effects cannot be excluded because the current rises were not fully reversible at higher menthol concentrations (> 100 μM). At these concentrations, an irreversible rise in intracellular Ca^2+^ levels occurred in human corneal endothelial cells (HCEC-12) and in TRPM8-overexpressing HEK293 cells (99) whereas in HCEC-12 a reversible effect was detectable (41). BCTC and AMTB are well-established TRPM8 antagonists, but AMTB is only TRPM8 selective whereas BCTC is also a TRPV1 antagonist (21, 24, 100). Nevertheless, both BCTC and AMTB had similar inhibitory effects on TRPM8 activation induced by either menthol or icilin (Figures 7–9) (98, 101). Specifically, icilin, which is a mixed TRPM8/ TRPA1 agonist (79) also irreversibly increased intracellular Ca^2+^ in HCEC and HCjEC (42–44) whereas these increases were also inhibited by AMTB confirming TRPM8 involvement in HCK (Figures 8D,G) (42–44). As icilin is both a super-cooling TRPM8 agonist, and a weak TRPA1 agonist (44, 102), its effects may include TRPA1 activation at either a high icilin/menthol concentration or cooling to ≈14°C (Figure 8A) (79). Nevertheless, the menthol/AMTB effects are very likely solely attributable to TRPM8 activation because icilin had effects that can only be accounted for by TRPM8 activation in mutant mice (98). In the current study, icilin induced increases in the intracellular Ca^2+^ levels and the whole-cell currents which were inhibited by both BCTC and AMTB (Figure 9). These inhibitory effects were similar to those occurring in HCEC (42). 3-T_1_AM was used as probe of thermo-sensitive TRPM8 involvement in mediating temperature lowering in rodents (20). Even though the responses induced by this thyroxine metabolite also included an interaction of TRPM8 with a GPCR in some other cell types, we did not deal with this possibility (4).

### VEGF interacts with TRPV1

The Ca^2+^transients and underlying ionic currents induced by VEGF are similar to those in microvascular endothelial cells and podocytes in which these responses were instead mediated by crosstalk with TRP canonical 6 (TRPC6) rather than TRPV1 (103, 104). In HCK, TRPV1 is solely involved because CPZ completely blocked these effects whereas AMTB had no effect (Figure 10). Unlike CPZ, AMTB instead slightly enhanced VEGF-induced whole-cell current, which supports the notion of TRPM8 suppressing crosstalk between TRPV1 and VEGFR (Figures 11D,E,F). The time delays seen in some cases in VEGF-induced rises in whole-cell currents and Ca^2+^ transients are probably due to drug diffusion delays (Figure 11A).

### 3-T_1_AM involvement in VEGF signaling

In this study, 3-T_1_AM blunting of the crosstalk between VEGFR and TRPV1 is similar to its inhibitory effect on TRPV1 activation by CAP in HCEC and HCjEC (42, 43). Other evidence supportive of crosstalk among these receptor triad members stems from the fact that AMTB increased inward currents induced by VEGF (Figures 11D,E,F) whereas 3-T_1_AM blunted VEGF-induced increases in whole-cell currents using a voltage ramp protocol (Figures 12A,B) or a voltage step protocol (Figures 12C,F). The current voltage relationships are consistent with TRP behavior described in previous studies of corneal cells (e.g., reversal potential, outwardly rectifying currents) (e.g., 13, 60). Interestingly, 3-T_1_AM had a delayed response which may be possibly attributable to a diffusion delay to an intracellular binding site. Another possibility requiring further study is that 3-T_1_AM instead indirectly targets TRPV1 via directly promoting the inhibitory effect of the (soluble) VEGF receptor 1 on VEGFR, which is also expressed in the cornea (55, 105). Such control by TRPM8 can be even more complex and may also involve interactions with adrenergic receptors (106).

### Potential therapeutic targets

This study provides additional supportive evidence that TRPV1 is a potential drug target for improving treatment of VEGF-induced neovascularization. Such an effect may be obtainable by suppressing VEGFR crosstalk with TRPV1 through increasing TRPM8 activity with an agonist such as thyronamine (3-T_1_AM). This strategy may provide a selective approach to inhibit TRPV1 upregulation and activation, which in turn suppresses angiogenesis, fibrosis as well as inflammatory processes in different pathophysiological conditions such as dry eye disease (DED), pterygium, and conjunctivitis sicca or red eyes (16, 94, 107). 3-T_1_AM may also be beneficial in the treatment of DED (42, 43, 49) since TRPM8 activation triggers increases in tear fluid production by the lacrimal gland via activation of the Central Nervous System (CNS) (40). In this context, borneol is another TRPM8 agonist, which provided symptomatic relief to DED patients when it was applied as eye drops (108–110).

## Conclusions

VEGF-induces Ca^2+^ transients by transactivating TRPV1, whereas TRPM8 activation suppresses this response by blocking TRPV1 activation. Accordingly, VEGF-induced corneal neovascularization may be inhibited by novel TRPM8 agonists such as 3-T_1_AM.

## Author contributions

SM, ErT, and PR designed the study, analyzed the data, wrote, and edited the manuscript. FG and TB performed immunofluorescence analysis and keratocyte validation. FG managed primary human corneal fibroblast cell culture. JK contributed with his expertise on thyroid hormone metabolites, discussed data and their interpretation, and helped edit the manuscript. UP also contributed with his expertise in medical issues and helped edit the manuscript. NK performed PCR analysis and immunohistochemistry. ErT, SM, AL, PJ, EliT, DC, FS, RR, and NL performed calcium measurements and planar patch-clamp recordings as well as plot analyses. ErT, PJ, NK, AL, EliT, and SM created diagrams.

### Conflict of interest statement

The authors declare that the research was conducted in the absence of any commercial or financial relationships that could be construed as a potential conflict of interest.

## Abbreviations

3-T_1_AM: 3-Iodothyronamine [endogenous thyroid hormone (TH)-derived metabolite (20)]
AMTB: N-(3-Aminopropyl)-2-[(3-methylphenyl)methoxy]-N-(2-thienylmethyl)benzamide hydrochloride [TRPM8 blocker (21)]
BCTC: N-(4- tertiarybutyl-phenyl)-4-(3-chloropyridin-2-yl) tetrahydropyrazine-1(2H)-carboxamide [TRPM8/TRPV1 inhibitor (22: 23)]
BPE: Bovine pituitary extract
BSA: Bovine serum albumin
CAP: Capsaicin [TRPV1 agonist (24)]
CD90: *C*luster of *D*ifferentiation 90 (25 26)
CNV: Corneal neovascularization
CPZ: Capsazepine [TRPV1 antagonist (24)]
DED: Dry eye disease
DMEM: Dulbecco's modified eagle's medium
EGF: Epidermal growth factor
FBS: Fetal bovine serum
HCCS: Human corneal cross sections
HCEC: Human corneal epithelial cells (27)
HCEC-12: Human corneal endothelial cells (28)
HCjEC: Human conjunctival epithelial cells (29)
HCK: Human corneal keratocytes (30)
hTCEpi: Telomerase-immortalized human corneal epithelial cells (31)
KGM®: *K*eratinocyte *B*asal *M*edium
KTN: Keratocan [major keratan sulfate (KS) proteoglycans in corneal stroma. (32 33)
LNCaP: *L*ymph *N*ode *Ca*rcinoma of the *P*rostate [human prostate adenocarcinoma cells (34)]
LUM: Lumican [major keratan sulfate (KS) proteoglycans in corneal stroma (32 33)]
MSC: Mesenchymal stem cells;
pHCF: Primary human corneal fibroblast cells
TBS: Tris-buffered saline
TGF β-1: Transforming growth factor β-1
TH: Thyroid hormone
TRPA: Transient receptor potential ankyrin
TRPC: Transient receptor potential canonical
TRPM: Transient receptor potential melastatin
TRPs: Transient receptor potential channels
TRPV: Transient receptor potential vanilloid
VEGFR: Vascular endothelial growth factor receptor
α-SMA: Alpha-smooth muscle actin (35 36).
